# Intrabasal Plane Defect Formation in NiFe Layered Double Hydroxides Enabling Efficient Electrochemical Water Oxidation

**DOI:** 10.1021/acsami.3c11651

**Published:** 2023-11-10

**Authors:** Xiaopeng Huang, Keon-Han Kim, Haeseong Jang, Xiaonan Luo, Jingfang Yu, Zhaoqiang Li, Zhimin Ao, Junxin Wang, Hao Zhang, Chunping Chen, Dermot O’Hare

**Affiliations:** † Department of Chemistry, Faculty of Arts and Sciences, Beijing Normal University, Zhuhai 519087, China; ‡ Chemistry Research Laboratory, Department of Chemistry, University of Oxford, 12 Mansfield Road, Oxford OX1 3TA U.K.; § Beamline Research Division, Pohang Accelerator Laboratory (PAL), Pohang 37673, Republic of Korea; ∥ Department of Materials, University of Oxford, 16 Parks Road, Oxford OX1 3PH, U.K.; ⊥ Engineering Research Center of NanoGeomaterials of Ministry of Education, China University of Geosciences, Wuhan 430074, China; # Faculty of Materials Science and Chemistry, China University of Geosciences, Wuhan 430074, China; ∇ Laboratory of Beam Technology and Energy Materials, Advanced Institute of Natural Sciences, Beijing Normal University, Zhuhai 519087, China; ○ Institute of Environmental Health and Pollution Control, School of Environmental Science and Engineering, Guangdong University of Technology, Guangzhou 510006, China; ◆ Advanced Interdisciplinary Institute of Environment and Ecology, Beijing Normal University, Zhuhai 519087, China; ¶ Department of Materials Science and Metallurgy, University of Cambridge, 27 Charles Babbage Road, Cambridge CB3 0FS, U.K.

**Keywords:** nanoplatelets, defect engineering, multiple vacancies, layered double hydroxides, oxygen reaction evolution

## Abstract

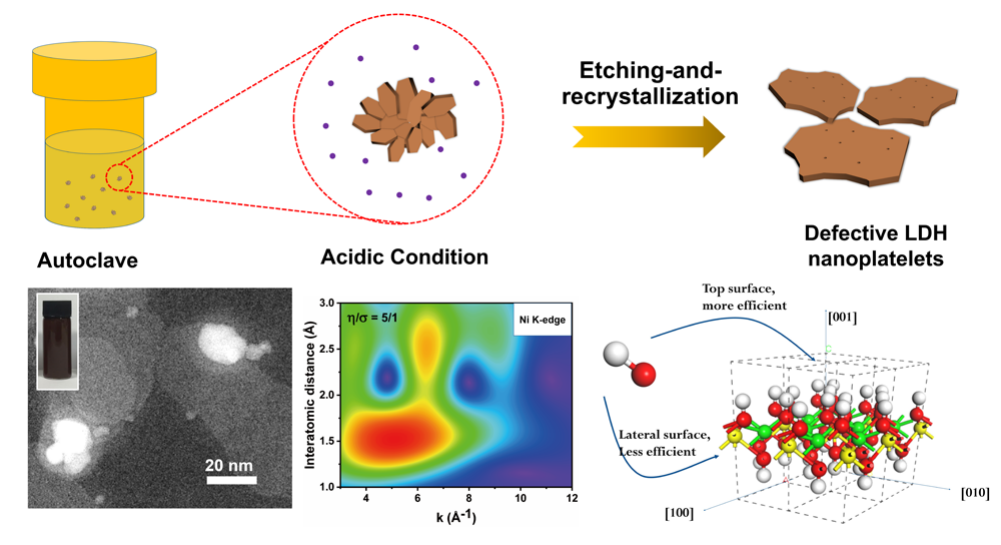

Defect engineering has proven to be one of the most effective approaches for the design of high-performance electrocatalysts. Current methods to create defects typically follow a top-down strategy, cutting down the pristine materials into fragmented pieces with surface defects yet also heavily destroying the framework of materials that imposes restrictions on the further improvements in catalytic activity. Herein, we describe a bottom-up strategy to prepare free-standing NiFe layered double hydroxide (LDH) nanoplatelets with abundant internal defects by controlling their growth behavior in acidic conditions. Our best-performing nanoplatelets exhibited the lowest overpotential of 241 mV and the lowest Tafel slope of 43 mV/dec for the oxygen evolution reaction (OER) process, superior to the pristine LDHs and other reference cation-defective LDHs obtained by traditional etching methods. Using both material characterization and density functional theory (DFT) simulation has enabled us to develop relationships between the structure and electrochemical properties of these catalysts, suggesting that the enhanced electrocatalytic activity of nanoplatelets mainly results from their defect-abundant structure and stable layered framework with enhanced exposure of the (001) surface.

## Introduction

In the past ten years, there has been a significant interest in designing high-performance electrocatalysts for the oxygen evolution reaction (OER) due to the demand of producing green hydrogen by water electrolysis. The OER is a critical half-reaction in the water electrolysis process as it is the efficiency-limiting step due to the multielectron transfer process and the resulting sluggish kinetics.^1−3^ The traditional benchmark OER catalysts are mainly noble metal-based oxides, such as ruthenium oxide (RuO_2_) and iridium oxide (IrO_2_); their development is limited due to their low reserves and high cost.^4,5^ Some of the most promising emerging candidate OER catalysts are transition metal-based layered double hydroxides (LDHs), such as NiFe LDHs, CoFe LDHs, and NiCo LDHs.^6−10^ LDHs are a family of two-dimensional layered materials; the most common examples have the general formula [M_1–*x*_^II^M_*x*_^III^(OH)_2_](A^*n*–^)_*x*/*n*_·*y*H_2_O, where M^II^ and M^III^ are divalent and trivalent metallic cations, respectively, and A^*n*–^ represents the intercalated charge-compensating counteranions.^10,11^ The use of the more earth-abundant transition metals in different combinations in the LDH framework can create synergistic effects that may improve electrocatalytic activity. Recent studies have shown that LDHs have excellent OER activity when compared to materials such as high-entropy alloys, spinels, and perovskites.^12^

The electrocatalytic activity of a catalyst is intimately related to its morphological, crystallographic, and electronic structure. As a result of the layered structure and electrical insulating nature, LDHs typically contain a low concentration of active sites and exhibit low electrical conductivity; hence, it is important to develop innovations to further improve their OER performance. Defect engineering has proved to be one of the most effective strategies to modulate the structure of materials for enhanced OER activity. It can regulate their electronic configuration, create more active sites, improve their conductivity, and thus increase their intrinsic activity.^13−15^ Several physical etching methods have been proposed to create defects, particularly cationic and anionic vacancies. Wang et al. employed argon plasma etching to introduce metal and oxygen vacancies in CoFe LDHs, creating an electrocatalyst with an OER overpotential as low as 266 mV at a current density of 10 mA/cm^2^.^16^ Zhou et al. reported a flame engraving method to create oxygen vacancies in NiFe DHs; this LDH displays an OER overpotential of ca. 270 mV.^17^ Yuan et al. prepared NiFe LDHs with multiple vacancies using a reconstruction method that exhibited an outstanding overpotential of ca. 370 mV in neutral media.^18^ However, these physical etching methods are typically hindered by complexity, cost, yield, and the challenges of scale-up. In contrast, chemical etching methods are more promising for practical application in view of the simple protocol and potentially low cost of scale-up. Both Zhou et al. and Peng et al. have used nitric acid to etch CoFe LDHs and NiFe LDHs, respectively; these samples reached an overpotential decrease from 346 to 300 mV for CoFe LDHs and from 342 to 308 mV for NiFe LDHs.^19,20^ Wang et al. selectively dissolved the Zn and Al cations from NiZnFe and NiFeAl LDHs, respectively, using strong alkali, eventually reaching ca. 14% lower overpotential compared to pristine NiFe LDH.^21^

One characteristic of these etching methods is the top-down strategy; this approach aims to break down a well-formed catalyst particle into a smaller, shorter, and more “defective” structure, leaving unsaturated metal cations on surfaces and edge sites or surface defects. These surface and edge site are high-energy states; their inherent instability will inevitably impact the improvement of the OER activity.^22^ Better strategies should be able to endow the catalyst with a high concentration of catalytically active internal defects while retaining a stable framework. In this study, we have evolved from a traditional chemical etching method to develop a bottom-up strategy using an etching-and-recrystallization method to synthesize LDHs with multiple internal defects and a stable layered framework motif. To better compare with previously reported studies, we choose NiFe LDHs as the reference samples. This method allows the efficient formation of a high defect concentration during the growth of the nanomaterials. Fine tuning of the etching and recrystallization steps leads to LDHs with superior OER activity and stability. Our material characterization and density functional theory (DFT) simulation experiments have enabled us to develop relationships between the structure and electrochemical properties of the catalysts.

## Methods

### Materials

Nickel nitrate hexahydrate (Ni(NO_3_)_2_·6H_2_O), iron nitrate nonahydrate (Fe(NO_3_)_3_·9H_2_O), sodium carbonate (Na_2_CO_3_), sodium hydroxide (NaOH), nitric acid (68%), ruthenium oxide (RuO_2_), and Nafion 117 solution (∼5% in a mixture of lower aliphatic alcohols and water) were purchased from Sigma-Aldrich. Nickel foam (1.6 mm in thickness) was received from MTI Corporation. Deionized water (15 MΩ/cm) was obtained from a Milli-Q water purification system. All chemicals were used without further purification.

### Synthesis of the Pristine Ni_3_Fe LDHs (NiFe Control)

The pristine Ni_3_Fe LDH, labeled as NiFe Control, was synthesized by a classical pH-controlled coprecipitation and centrifugation protocol. Typically, 37.5 mmol Ni(NO_3_)_2_·6H_2_O and 12.5 mmol Fe(NO_3_)_3_·9H_2_O were mixed and dissolved in 50 mL of water, labeled as solution A. Solution B is prepared by dissolving 100 mmol NaOH in 100 mL of water (1 M). Solution C is obtained by dissolving 6.25 mmol Na_2_CO_3_ in 50 mL of water. Then, solution A is injected into solution C in a beaker using a syringe pump at a flow speed of 115 mL/h, and the reaction was kept at a stirring speed of 800 rpm. The pH of the solution was monitored using a pH meter (Mettler Toledo) and controlled to be 10 by dropwise adding solution B manually. A yellow-green precipitate of Ni_3_Fe LDH was observed once the reaction started. After a total reaction time of 1 h, around 200 mL of the suspension (∼500 mg samples) was divided into 8 parts, followed by a centrifugation–redispersion protocol using deionized water three times, which was the first centrifugation–redispersion protocol. The precipitates were labeled as LDH seeds, which were dispersed in 30 mL of water and aged for 20 h at room temperature under a stirring speed of 100 rpm. Finally, after a second centrifugation–redispersion protocol one time, all the samples are collected for further characterization.

### Synthesis of the LDHs by an Etching Method of NiFe; *E*_*y*_ (*y* = 1–10)

The initial synthesis of these NiFe *E*_*y*_ LDHs by an etching method is similar to that of pristine LDHs. The difference is that after the first centrifugation–redispersion protocol, each sample was mixed with different amounts of nitric acid solution (0.9 M), i.e., 1, 2, 5, 7.5, and 10 mL for NiFe E1, NiFe E2, NiFe E5, NiFe E7.5, and NiFe E10, respectively. The nitric acid treatment was left for 20 h at room temperature (25 °C) for all the samples and at 50 °C for NiFe E5. Finally, the second centrifugation–redispersion/washing protocol was performed three times to neutralize the sample.

### Synthesis of the LDHs by an Etching-and-Recrystallization (ER) Method of NiFe; ER_*y*_ (*y* = 0–10)

The initial synthesis of these NiFe ER_*y*_ LDHs by an ER method is similar to that of pristine LDHs. The difference is that after the first centrifugation–redispersion protocol, each sample was mixed with different amounts of nitric acid solution (0.9 M), ie. 0, 1, 2, 5, 7.5, and 10 mL for NiFe ER1, NiFe ER2, NiFe ER5, NiFe ER7.5, and NiFe ER10, respectively. Then, the samples were transferred to a Teflon-lined stainless-steel autoclave and heated at a hydrothermal temperature of 100 °C for 20 h. NiFe ER0 was hydrothermally treated without adding acid. For the NiFe ER5, hydrothermal temperatures of 150 and 200 °C were also used for comparison. Finally, the second centrifugation–redispersion/washing protocol was performed three times to neutralize the sample.

### Characterization

Scanning electron microscopy (SEM) images were taken on Carl Zeiss Merlin field emission scanning electron microscopes under in-lens mode operating at an accelerating voltage of 5 kV. Transmission electron microscopy (TEM) graphs, scanning transmission electron microscopy (STEM) images, and energy-dispersive X-ray spectroscopy (EDX) images were captured using a JEOL ARM 200F electron microscope fitted with a probe-forming aberration corrector; prior to that, the LDH materials were dispersed in ethanol using sonication and dropped and dried on ultrathin carbon-supported copper grids. Powder X-ray diffraction (XRD) measurement was performed on a PANAnalytical X’Pert Pro X-ray powder diffractometer. Fourier-transform infrared spectroscopy (FTIR) spectra were recorded using a Bruker VERTEX 80 spectrometer equipped with a DuraSamplIR II diamond ATR (attenuated total reflection) accessory. The thermal gravimetric analysis (TGA) curve was measured using a Mettler Toledo TGA/DSC 1 system from 30 to 600 °C at a heating speed of 10 °C/min. The pH values were measured using a Mettler Toledo pH meter. The nitrogen adsorption and desorption isotherms were collected at 77 K on a Micromeritics TriStar II 3030 instrument, which were used to determine the specific surface area based on the Brunauer–Emmett–Teller (BET) method and pore size and volume based on the Barrett–Joyner–Halenda (BJH) model. The LDH samples were degassed at 110 °C overnight before analysis. Inductively coupled plasma optical emission spectrometry (ICP-OES) was used to analyze the nickel and iron elements using a PerkinElmer Optima ICP spectrometer in the University of Cambridge. The analysis of carbon, hydrogen, and nitrogen was performed on a Thermo Flash 2000 machine in London Metropolitan University. Atomic force microscopy (AFM) measurement was carried out using a NanoScope MultiMode atomic force microscope using tapping mode with a silicon tip coated with aluminum. The synchrotron EXAFS and XANES of Co and Fe K-edge were carried out at the 10C beamline of Pohang Accelerator Laboratory (PAL) in Pohang, Korea.

### Electrode Preparation

Two types of electrodes were prepared. The first one was based on a drop-casting method on a glassy carbon electrode. A glassy carbon electrode was thoroughly cleaned and polished to a mirror finish before use. An electrode ink was prepared by mixing 500 μL of the samples with 30 μL of Nafion solution and kept for sonication for at least 10 min. Finally, 5 μL of the ink (∼23 μg of the catalyst) was carefully dropped on the center of the glassy carbon electrode and dried in a vacuum oven to form a flat and intact catalyst film. The second one was based on a dip-coating way on nickel foams. A number of nickel foams, cut into 1 cm × 1 cm in size, were sonicated in acetone for 30 min and in deionized water for 10 min to remove surface organics, followed by drying in an oven at 60 °C overnight. Then, a nickel foam was immersed in a LDH catalyst suspension for 1 min and transferred to an oven to be dried at 110 °C for 1 h. Afterward, the nickel foam was sonicated in acetone for 60 min and in deionized water for 10 min two times to remove the LDH catalysts, which were not well adhered to the nickel foam. The coating procedure was repeated two or three times to get a catalyst loading of ∼3 mg in each nickel foam. Finally, the nickel foam with LDH coatings was used as the electrode. The preparation of the RuO_2_ electrode used similar procedures by replacing LDH nanomaterials with RuO_2_ particles; the suspension of RuO_2_ in water took at least 2 h for the sonication.

### Electrochemical Characterization

A Gamry Reference 3000 potentiostat was used to test all of the electrochemical properties. The test of the OER activity was performed in a three-electrode system, containing a glassy carbon (3 mm in diameter) with LDH catalysts as the working electrode, a Hg/HgO as the reference electrode, and a Pt wire as the counter electrode. The electrolyte was 1 M potassium hydroxide (KOH) solution saturated with nitrogen gas. All potentials obtained were converted to the reversible hydrogen electrode (RHE) according to *E*_RHE_ = *E*_Hg/HgO_ + 0.059 pH + 0.0977. Before all the tests, a cyclic voltammetry (CV) scan from 0.9 to 1.6 V was applied to activate the catalysts, which took at least 20 cycles until a stable current density was reached. The linear sweep voltammetry (LSV) scanning at a scanning speed of 5 mV/s was used as polarization curve. All the polarization curves were corrected by eliminating *iR* drop manually where *R* is the ohmic resistance of the solution. Electrochemical impedance spectroscopy (EIS) was performed under 1.5 V over the frequency range of 0.1–10^5^ Hz with an AC voltage of 10 mV. The CV scanning for the electrochemical double-layer capacitance was performed from 0.95 to 1.00 V at scanning speeds of 2.5, 5, 10, 20, and 40 mV/s. The test of the OER stability used the nickel foam coated with LDH catalysts as the electrode. After CV scanning until a stable current density was obtained, the chronopotentiometric test was performed under a constant current density of 100 mA/cm^2^.

### Calculation Methods

The density functional theory (DFT) calculation of the electrochemical free energy diagram was conducted in a Dmol3 package embedded in Materials Studios 7.0 (Accelrys Co.). All the models were calculated in a spin-unrestricted form, using generalized-gradient approximation (GGA) with the Perdew–Burke–Ernzerh (PBE) functional, together with a double numerical plus polarization (DNP) basis set and an effective core potential (ECP) treatment. An electronic self-consistent field tolerance of 10^–6^ Ha and a global orbital cutoff of 4.5 Å were used for the calculation. A smearing value of 0.01 Ha was adopted to speed up the convergence. For the geometry optimization, the convergence tolerance of energy is 10^–5^ eV per atom, and the maximum allowed displacement and force are 0.005 Å and 0.002 eV/Å, respectively.

We built slabs of the (001) surface and (100) surface of Ni_3_Fe LDHs in a vacuum box, where a spacing of 15 Å between the slabs was applied to avoid the interaction of neighboring slabs in the thickness direction. Each of the slab consisted of three Ni atoms, one Fe atom, eight O atoms, and seven H atoms, in which a H vacancy was created to keep charge neutrality. A k-point mesh of 3 × 3 × 1 was used for the energy calculation. During structure optimization, the absorbates involved in the OER were O*, OH*, and OOH*. In an alkaline environment, the critical four elementary steps of the OER process based on the adsorbate evolution mechanism can be written as follows
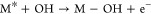
S1
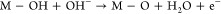
S2
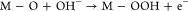
S3
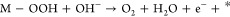
S4where M* represents the catalyst with an active adsorption site and M–OH, M–O, and M–OOH mean the catalyst with the corresponding absorbates. According to the computational hydrogen electrode model, Gibbs reaction free energy (Δ*G*) is defined as the difference between the initial and final energies, which is calculated by the following equation
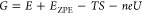
S5in which *E* is the structure energy, *E*_ZPE_ is the zero-point energy (calculated based on the vibration frequencies), *T* is the room temperature (298.15 K), *S* is the entropy, *n* is the number of proton–electron pairs transferred, *e* is the electron transferred, and *U* is the applied potential relative to the RHE (only *U* = 0 is considered in our calculation).

By setting the reference potential to be the standard hydrogen electrode, the free energy of (H^+^ + e^–^) was replaced by 1/2H_2_. At a standard condition, the reaction free energy for the four elementary steps can be calculated as follows
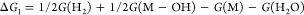
S6
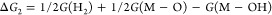
S7
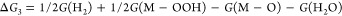
S8
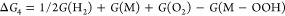
S9where *G*(O_2_), *G*(H_2_), and *G*(H_2_O) are the free energy of O_2_, H_2_, and H_2_O molecules in the gas phase, respectively. To make the free energy of the ground triplet state of O_2_ molecules (*G*(O_2_)) more reliable, *G*(O_2_) was calculated according to *G*(O_2_) = 2*G*(H_2_O) – 2*G*(H_2_) + 4.92 eV. Thus, Δ*G*_4_ can be rewritten as
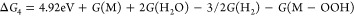
S10

Finally, the OER theoretical overpotential (η) of different reaction routes is determined by the following equation
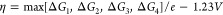
S11

## Results and Discussion

### Synthesis

**Scheme 1:**
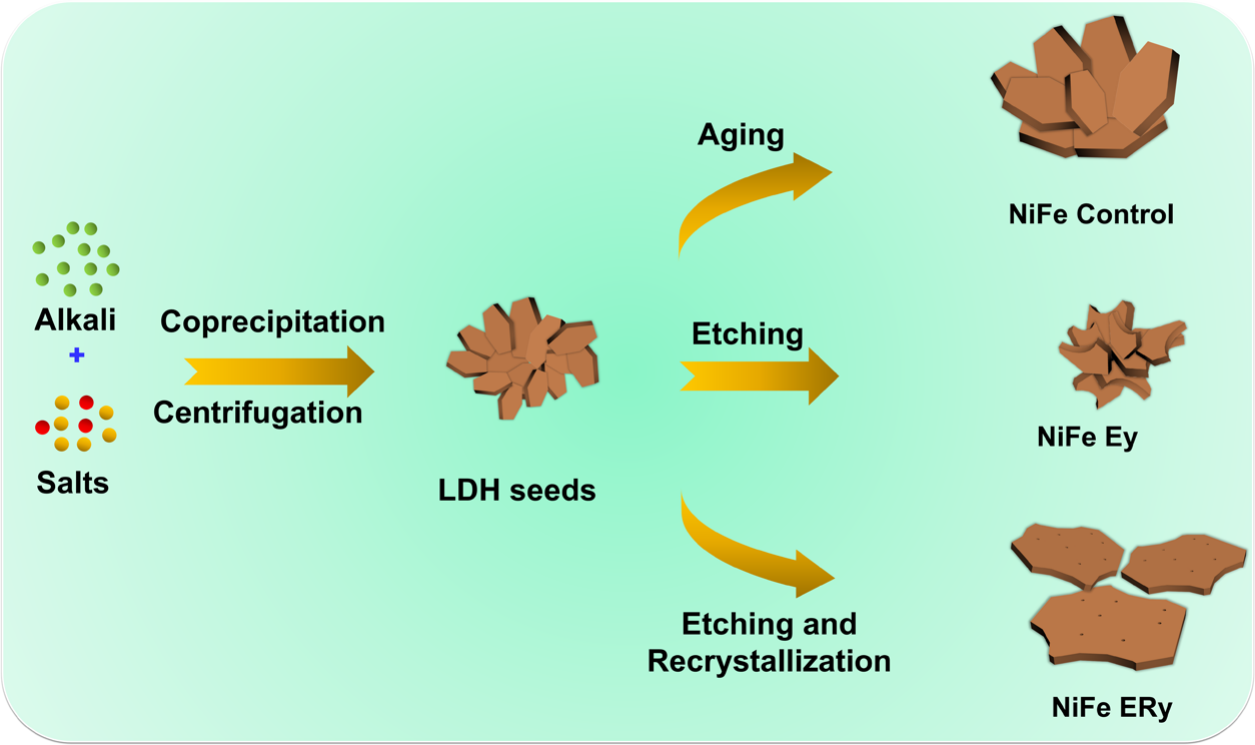
Synthesis Protocol for Three Types of LDHs: Aging, Etching, and Etching and Recrystallization

In this work, we have investigated a novel etching-and-recrystallization protocol as shown in Scheme 1. Initially, we synthesized Ni_3_Fe-CO_3_ LDH (idealized formula) seeds using pH-controlled coprecipitation, followed by aging at room temperature (RT) to form [Ni_0.76_Fe_0.24_(OH)_2_](CO_3_)_0.1_(NO_3_)_0.03_ as a control sample (labeled as Ni_3_Fe Control). Then, a series of defective LDH samples were prepared using two protocols. In the first series, similar to those published previously,^19,20^ Ni_3_Fe LDH seeds (500 mg) were exposed to various volumes of 0.9 M nitric acid at 25 or 50 °C for 20 h to give NiFe *E*_*y*_ LDH, where ‘*E*_*y*_’ refers to the volume of acid (y/mL). For example, NiFe E5 was obtained by adding 5 mL of 0.9 M nitric acid at room temperature or 20 h to 500 mg of Ni_3_Fe-CO_3_ LDH; this procedure produces [Ni_0.64_Fe_0.36_(OH)_2_](CO_3_)_0.02_(NO_3_)_0.33_. A second LDH series was characterized by exposing 500 mg of the Ni_3_Fe-CO_3_ LDH control seeds to various volumes of 0.9 M nitric acid under hydrothermal conditions (100–200 °C) for 20 h to give NiFe ER_*y*_ LDH, where ‘ER_*y*_’ refers to the volume (*y* mL) of nitric acid. For example, NiFe ER5 is obtained by adding 5 mL of 0.9 M nitric acid to 500 mg of Ni_3_Fe LDH followed by hydrothermal treatment at 100 °C. This produces a material with the composition [Ni_0.64_Fe_0.36_(OH)_2_](CO_3_)_0.07_(NO_3_)_0.21_ (XRD shows the presence of a small amount of Fe_3_O_4_) as determined using a combination of inductively coupled plasma optical emission spectrometry (ICP-OES) and CHN elemental analysis.

### Morphological and Structural Features

**Figure 1:**
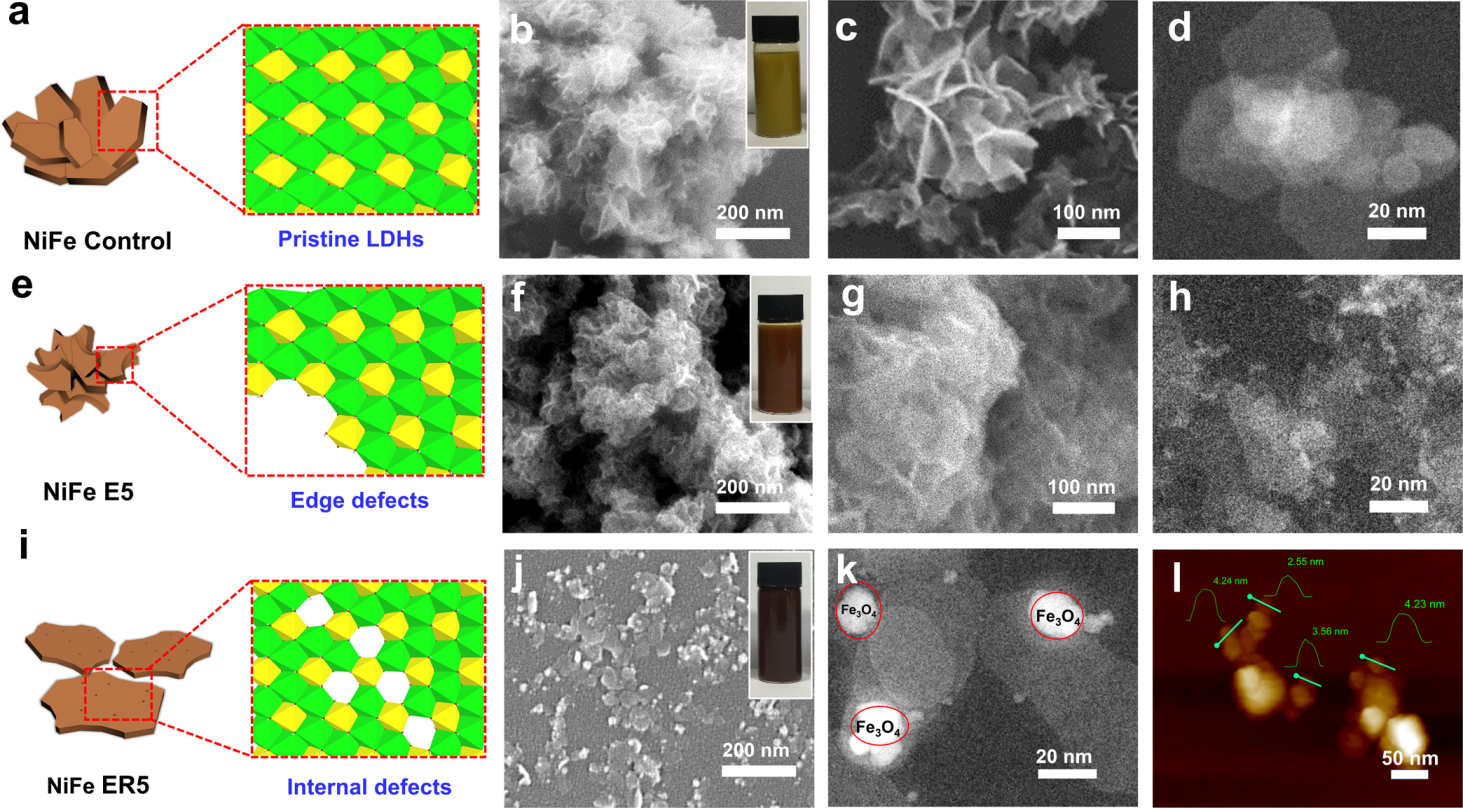
(a, e) (i) Schematic illustrations and (b, c, f, g, j) SEM, (d, h, k) STEM, and (l) AFM images of the LDHs synthesized by three different methods; (a–d) Ni_3_Fe LDH synthesized by coprecipitation and room-temperature aging, (e–h) NiFe E5 synthesized after etching at RT, and (i–l) NiFe ER5 synthesized by etching and recrystallization at 100 °C. Insets of (b, f, j) are the images of the samples dispersed in water.

As shown in Figure 1, scanning electron microscopy (SEM), scanning transmission electron microscopy (STEM), and atomic force microscopy (AFM) have been used to characterize the morphological structure of all the LDHs. Ni_3_Fe LDH (NiFe Control) exhibits flower-like nanoplatelet aggregates with a lateral dimension of 30–50 nm; these aggregates form as a result of the heterogeneous nucleation of nanoplatelets on the early formed nanoplatelets (Figure 1a–d).^23^ After etching, NiFe E5 retains its flower-like aggregate morphology, although its lateral size is reduced to less than 20 nm as some surfaces and edges were dissolved by the acid (Figure 1e–h). More substantial etching of the nanoplatelets was observed when using larger quantities of 0.9 M nitric acid (Figure S1); this is accompanied by the change in color from dark green to brown; the LDHs completely dissolved in 10 mL of 0.9 M nitric acid. NiFe ER5, the LDH obtained after etching and recrystallization, presents well-dispersed free-standing nanoplatelets with the lateral size of 20–50 nm as determined by the SEM, TEM, and AFM (Figures 1i–l and S2d); a platelet thickness of 2.5–4.5 nm can be determined from the AFM images (Figure 1l). The hydrothermal treatment allows the flower-like aggregates to dissolve and recrystallize, allowing the lateral growth of free-standing nanoplatelets.^24^ In the absence of acid during the hydrothermal treatment, well-dispersed LDH platelets (NiFe ER0 in Figure S2a) can also be formed, with lateral platelets more than 100 nm. We believe that the low-pH environment created by the nitric acid inhibits the precipitation of insoluble metal hydroxides that eventually directs LDH formation with smaller platelet diameters and thicknesses (Figure S2). On the other hand, hydrothermal treatment at 200 °C promotes recrystallization of the LDHs, leading to platelets with diameters of up to 200 nm (Figure S3). Hydrothermal treatment also leads to the formation of 5–20 nm magnetic iron oxide (Fe_3_O_4_) particles as shown in the SEM and STEM images (Figure 1j,k); the quantity of this impurity phase increases with increasing amount of nitric acid or hydrothermal processing temperature (Figures S2–S4). The formation of Fe_3_O_4_ nanoparticles was presumably due to the production of Fe(OH)_*x*_ fragments during the acid etching process, which then transformed into Fe_3_O_4_ nanoparticles after the hydrothermal process.

**Figure 2:**
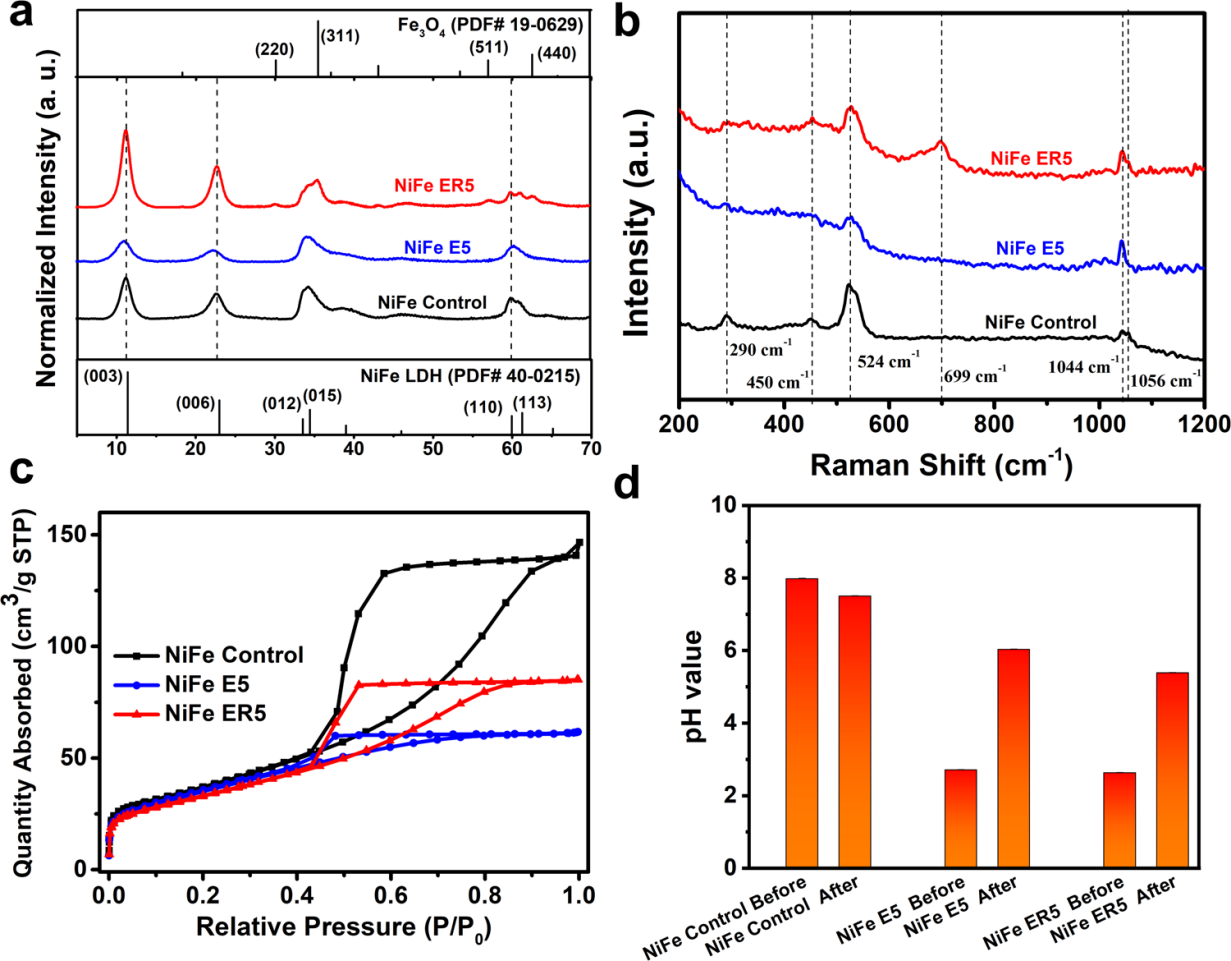
(a) XRD data, (b) Raman spectra, and (c) N_2_ adsorption/desorption isotherms of Ni_3_Fe LDH, NiFe E5, and NiFe ER5. (d) pH change before and after the reaction for 20 h.

The X-ray diffraction (XRD) data (Figure 2a) for Ni_3_Fe LDH present typical LDH Bragg reflections that may be indexed as (003), (006), (012), (015), (110), and (113). The calculated lattice parameters *a* = 0.308 nm and *c* = 2.334 nm (Table 1) agree well with bulk Ni_3_Fe-CO_3_ LDH (PDF 40-0215). After being etched at room temperature, the Bragg reflections of NiFe E5 are broader (Figures 2a and S5). Analysis of the crystal domain lengths (CDL), using the Scherrer equation, indicates a reduction in CDL both in the *ab*-plane and along the stacking axis (*c*-axis), which is in accordance with the morphological structures (Figure 1f–h). Similar results were found for the NiFe *E*_*y*_ samples, showing a decrease in the CDL as more nitric acid was added (Table S1). For NiFe ER5 obtained after etching and recrystallization, in the XRD data, we observe a narrowing of the Bragg reflections; the CDL in the ab-plane was determined to be 22.7 nm, almost twice the value compared to both Ni_3_Fe LDH (12.4 nm) and NiFe E5 (10.8 nm), which indicates the presence of a more crystalline layered structure for NiFe ER5 (Table 1).

**Table 1:** Lattice Parameters, Crystal Domain Length (CDL), Ni/Fe Molar Ratio, and C/N Ratio of Ni_3_Fe LDH, NiFe E5, and NiFe ER5

	lattice parameters (nm)^a^		crystal domain length (CDL) (nm)^b^			
	*a* (nm)	*c* (nm)	*ab*-plane	*c*-axis	Ni/Fe ratio (*x*)	C/N ratio
NiFe control	0.308	2.334	12.4	8.8	3.2	2.8
NiFe E5	0.306	2.379	10.8	8.3	1.8	0.1
NiFe ER5	0.308	2.346	22.7	9.6	1.8	0.4

a Lattice parameter (*a*) is calculated as *a* = 2*d*_110_ and (*c*) is calculated as *c* = 3*d*_003_.

b CDL is determined using the Scherrer equation, *D*_*hkl*_ = *R*λ/β cos* *θ, where *D*_*hkl*_ is the length in a specific crystallographic direction, *R* is the Scherrer constant (0.89), λ is the wavelength (0.1542 nm), β is the peak width at half-maximum (rad), and θ is the Bragg angle (rad).

Small changes are also observed for the interlayer spacing (*c*-axis lattice parameter); the interlayer spacing for NiFe ER5 is larger than that of Ni_3_Fe LDH yet smaller than that of NiFe E5. Table S2 indicates that in the NiFe ER_*y*_ series, adding more 0.9 M nitric acid leads to an increase in the interlayer spacing and a decrease in the CDL. However, the increase in the interlayer spacing for NiFe ER_*y*_ is smaller than that for NiFe *E*_*y*_, and their CDL in *ab*-plane is much larger (Table S3), suggesting that NiFe ER_*y*_ samples have higher crystallinity than that of the NiFe E_*y*_ samples. Further increasing the content of 0.9 M nitric acid leads to a decrease in the intensity of any LDH Bragg reflection and an increase in the intensity of reflections due to impurity phases; eventually, no LDH forms when using 10 mL of 0.9 M nitric acid (Figure S6). The LDH crystallinity remains constant with increasing hydrothermal treatment up to 200 °C (Figure S7). Overall, the optimal use of acid amount and hydrothermal processing temperature are two important factors in this etching–recrystallization process.

The Ni/Fe (*x*) ratio in NiFe E5 and NiFe ER5 is ca. 1.8 as determined by ICP-OES; the Ni/Fe ratio in the Ni_3_Fe Control was found to be 3.2 (Table 1). The analytical data indicate that the Ni cations are preferentially lost in the acidic etching process compared to the Fe cations. The defective structure of these LDHs can also be observed using Raman spectroscopy (Figure 2b). Ni_3_Fe LDH shows characteristic resonances at 290, 450, and 524 cm^–1^, assigned as E-type, M–O(H), and M–O vibrations, respectively.^25^ In both NiFe E5 and NiFe ER5, these resonances are broadened and less intense, suggesting a more disordered local environment for M–O(H) and M–O.^26^ Addition of more nitric acid, regardless of the temperature (Figures S8 and S9), results in the decrease and eventual disappearance of the LDH-derived resonances, which is in agreement with the XRD data. At higher temperature, additional resonances at 325, 475, and 699 cm^–1^ (Figure S10) are observed that are assigned to the *E*_g_, *T*_2g_, and *A*_1g_ modes of Fe_3_O_4_ nanoparticles, respectively.^27^ These features become predominant at 200 °C, indicating the presence of a large amount of crystalline Fe_3_O_4_ nanoparticles. Additionally, the resonances at 1044 and 1056 cm^–1^ are assigned to the characteristic vibrations of nitrate and carbonate, respectively. It is found that the carbonate vibration becomes weaker, while the nitrate vibration becomes predominant with increasing amount of nitric acid (Figure S9). This is consistent with anion exchange of carbonate with nitrate during an etching environment using nitric acid. Autogenous hydrothermal conditions appear to favor the retention of the intercalated carbonate during etching, as observed by the CHN elemental analysis showing a high C/N ratio in NiFe ER5 and a low C/N ratio in NiFe E5 (Table 1). Thermogravimetric analysis (TGA) data for Ni_3_Fe LDH (Figure S11) indicate that these LDHs have three weight loss events upon heating, including loss of surface-adsorbed and interlayered water from 30 to 220 °C, loss of hydroxyl groups from 220 to 300 °C, and decomposition of the interlayer anions from 220 to 500 °C. The LDHs obtained by the etching and recrystallization exhibit less dehydration and less dehydroxylation, which is due to the reduced amount of LDHs in these samples. The surface area and porosity of the three types of the LDH sample were studied using nitrogen adsorption/desorption experiments. The adsorption and desorption curves show that all the LDHs exhibit a type IV isotherm with a H2 hysteresis shape according to the IUPAC classification (Figure 2c).^28^ The NiFe Control has the largest BET-derived specific surface area (133.4 m^2^/g), while the NiFe E5 and ER5 have a similar surface area of ca. 120 m^2^/g (Figure S12a). The hysteresis loops at a relative pressure of above 0.4 *p*/*p*_0_ are caused by the capillary condensation due to the mesoporous structure. The hysteresis loops are observed in the order Ni_3_Fe LDH > NiFe ER5 > NiFe E5, which is consistent with the trends in the pore diameter and pore volume (Figure S12); this further supports the conclusion that these LDHs lose more nanostructured crystallinity after acid etching at RT than after the etching-and-recrystallization treatment. The pH of the reaction medium before and after the treatment was monitored; a decrease in pH means that some hydroxyl ions are used to make the LDHs, while an increase in pH would suggest dissolution of the LDHs by acid etching and release of hydroxyl ions into the solution phase. The pH of the LDH suspension after coprecipitation before aging is ca. 8.0. It decreased to ca. 7.5 after aging for 20 h without acid, indicating that the aging process helps in the further growth of LDHs (Figures 2d and S13). In the presence of acid (0.9 M, 5 mL), the pH of the LDH suspension rapidly dropped to ca. 2.7 and then increased to ca. 6.0 after etching at RT for 20 h (Figure 2d). However, after the etching-and-recrystallization process at 100 °C for 20 h, the pH only increased to ca. 5.3, indicating that less LDHs are etched due to the hydrothermal conditions.

### Vacancy Characterization

**Figure 3:**
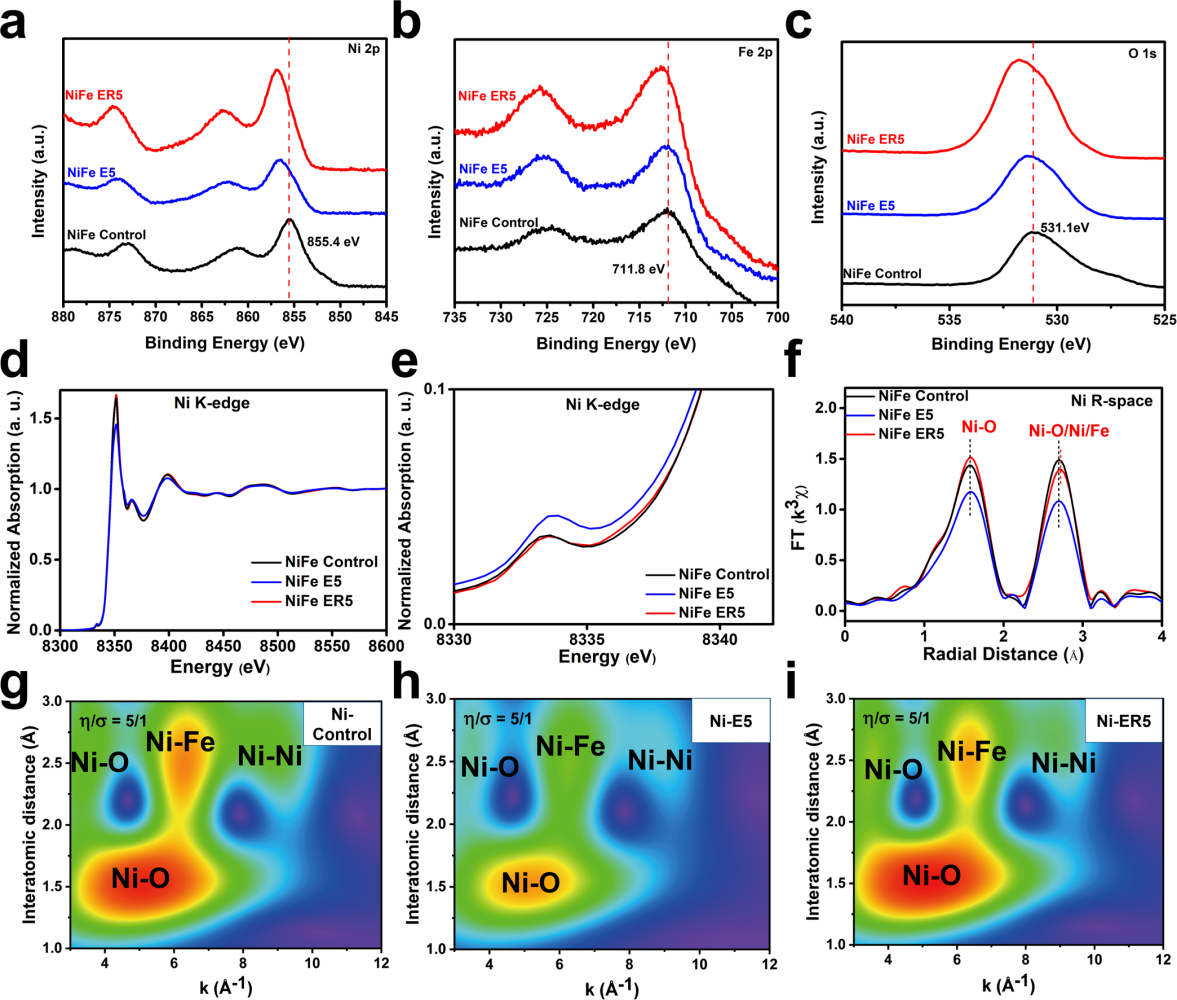
(a–c) XPS spectra of Ni_3_Fe LDH, NiFe E5, and NiFe ER5. Ni 2p (a), Fe 2p (b), and O 1s (c), (d, e) Ni K-edge XANES spectra in a full (d) and enlarged view (e), (f) *k*^3^-weighted Fourier-transformed Ni K-edge EXAFS data of Ni_3_Fe LDH, NiFe E5, and NiFe ER5, and (g–i) wavelet-transformed Ni K-edge EXAFS analysis of Ni_3_Fe LDH (g), NiFe E5 (h), and NiFe ER5 (i).

X-ray photoelectron spectroscopy (XPS) experiments were carried out to further understand the surface defects in the three types of LDH samples. As shown in Figure 3a,b, the Ni 2p and Fe 2p photoemission peaks of NiFe ER5 shift to the high-energy direction relative to both Ni_3_Fe LDH and NiFe E5, implying that the metal cations on the surface of NiFe ER5 have a higher oxidation state. Further analysis was obtained by deconvolving the photoemission feature of Ni 2p_3/2_ into photoemission peaks due to Ni^2+^ 2p_3/2_ at 855.3 eV and Ni^3+^ 2p_3/2_ at 856.9 eV and the photoemission peak of Fe 2p_3/2_ into photoemission features of Fe^2+^ 2p_3/2_ at 709.5 eV, Fe^3+^ 2p_3/2_ at 711.5 eV, and Fe^4+^ 2p_3/2_ at 714.1 eV (Figures S14 and S15).^29−32^ The formation of metal vacancies allows the remaining metal cations to share more oxygen ions, thus making more higher valence (Ni^3+^ and Fe^4+^) species within the LDH as a result of charge compensation. NiFe ER5 clearly exhibits the highest proportion of Ni^3+^ 2p photoemission peaks and Fe^4+^ 2p photoemission peaks, suggesting that more Fe and Ni vacancies are created by the etching-and-recrystallization process. The O 1s photoemission peak from NiFe ER5 was also observed to shift to higher binding energy. The deconvolution of the O 1s photoemission peak can be assigned to emission from nitrate at 532.8 eV, carbonate at 530.7 eV, metal-hydroxyl (M–O–H) at 531.2 eV, water (H–O–H) at 532.6 eV, metal–oxygen (M–O–M) at 529.4 eV, lattice oxygen vacancies at 531.6 eV, and iron–oxygen (Fe–O) in Fe_3_O_4_ nanoparticles at 530.5 eV (Figure S16).^33,34^ The fitted occupation of lattice oxygen vacancies in NiFe ER5 is 33.3%, higher than 28.0% in NiFe E5 and 3.6% in Ni_3_Fe LDH, demonstrating that NiFe ER5 contains the highest proportion of oxygen vacancies. Due to the extended lateral dimensions of NiFe LDHs, these defects are located inside the basal (001) planes rather than as dangling bonds on the edge surface.

The local structural environment of the samples was further analyzed using both X-ray absorption near-edge structure (XANES) and extended X-ray absorption fine structure (EXAFS) techniques (Figure 3d–i). In contrast to XPS, which is a more surface-sensitive analytical technique, XANES and EXAFS provide a bulk analytical insight. Figure 3e shows that the Ni K-edge pre-edge feature for NiFe E5 shifts to the low-energy direction relative to that of Ni_3_Fe LDH, while the Ni K-edge pre-edge for NiFe ER5 remains the same as in Ni_3_Fe LDH. We believe that this can be rationalized by the balancing of the cationic and anionic vacancies in NiFe ER5, while for NiFe E5, net oxygen vacancies result in a lower average Ni oxidation state after charge compensation. Similarly, the Fe K-edge XANES spectra of NiFe E5 shift to lower energy compared with those of Ni_3_Fe LDH, consistent with the presence of excessive oxygen vacancies (Figure S17b). The Fe K-edge XANES spectra of NiFe ER5 are also consistent with an overall more reduced valence state (perhaps from divalent Fe species in the impurity Fe_3_O_4_ nanoparticles). Fourier-transformed Ni K-edge EXAFS data indicated that compared to Ni_3_Fe LDH, NiFe E5 has a noticeably weaker intensity in both the first shell (assigned to Ni–O scattering) and the second shell (assigned to Ni–O scattering from the adsorbed anions, Ni–Fe scattering, and Ni–Ni scattering), indicating a lower coordination number due to the creation of oxygen and metal defects; these observations are in agreement with other earlier reports (Figure 3f).^16,18,19^ The contributions from different scattering paths can be additionally distinguished by the wavelet-transformed EXAFS analysis, which clearly indicates that all the interactions become weaker in NiFe E5 compared with those in Ni_3_Fe LDH, confirming the reduced coordination number of Ni–O, Ni–Fe, and Ni–Ni environments or the existence of substantial O, Fe, and Ni defects (Figure 3g,h).^35,36^ Similar results can also be found in the Fe K-edge EXAFS analysis (Figure S17c–e). While for NiFe ER5, it shows an increase in the first shell and a decrease in the second shell (Figure 3f). In principle, it should show a weaker intensity in both coordination shells in view of the existence of more surface vacancies as detected by XPS. However, the decrease in the degree of disorder due to high-temperature recrystallization simultaneously leads to an increase of the intensity in the *R* space of the EXAFS.^37^ The enhancement of the Ni–O scattering path demonstrates that even though NiFe ER5 has more vacancy defects, it is overall more crystalline. The decrease of the second shell may be further investigated by wavelet-transformed EXAFS analysis. It is found that the Ni–O scattering from the adsorbed anions increases, while both Ni–Fe scattering and Ni–Ni scattering are reduced; this is consistent with the former having a larger plate-like morphology, benefiting the adsorption of anions, while the latter is the result of more Ni and Fe vacancies (Figure 3i). The Fe EXAFS of NiFe ER5 shows a decrease in the first and second shells, which may be a convolution of effects from both the LDH and the Fe_3_O_4_ nanoparticles (Figure S17c,f). These findings additionally support the hypothesis that by tuning the nitric acid treatment and hydrothermal processing temperature in the etching-and-recrystallization route, the morphology, crystallinity, and the local defect state of these LDHs can be adjusted via a balance of etching and growth processes. Overall, we find that this is highly beneficial for the optimization of their electrocatalytic activities (vide infra).

### Electrocatalytic Performance

**Figure 4:**
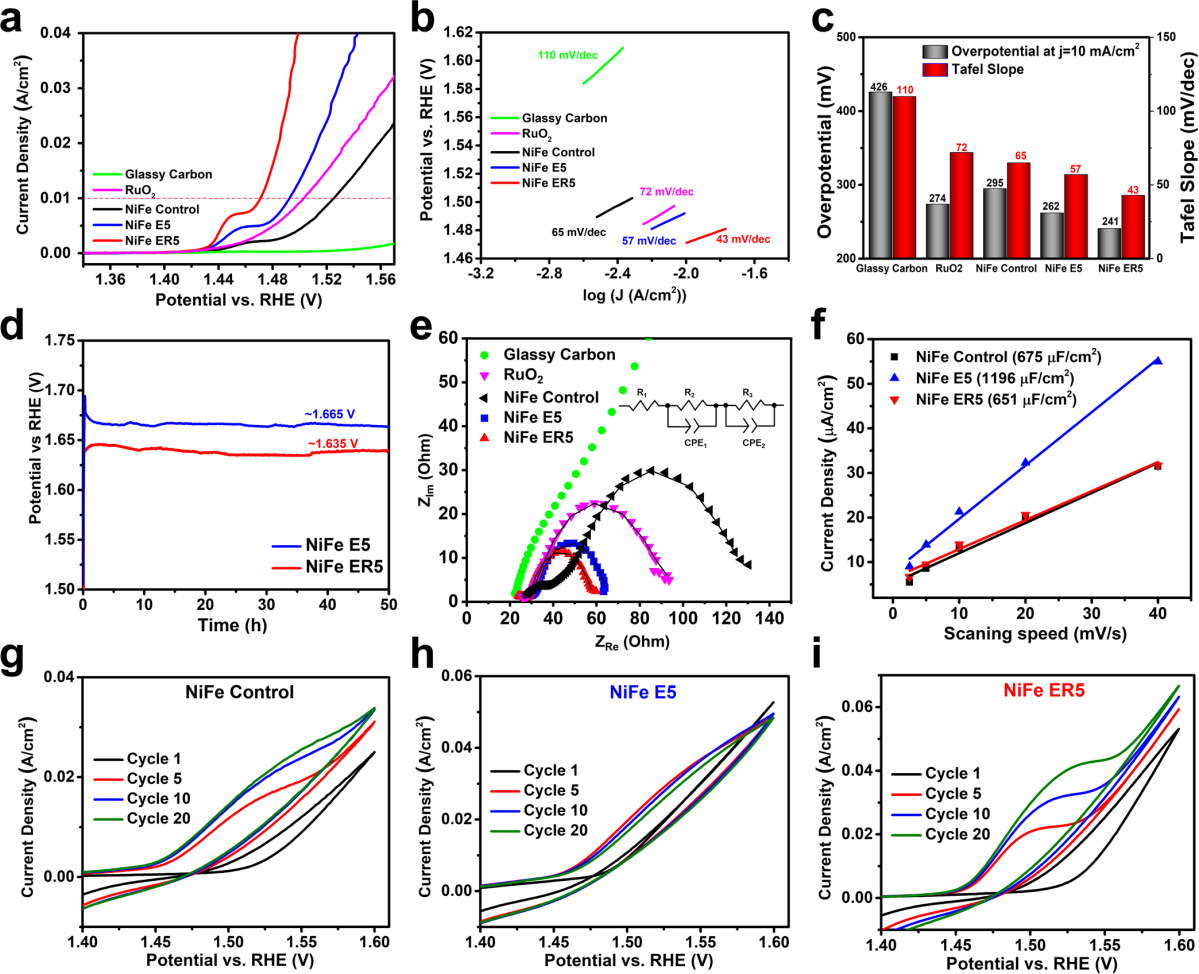
OER performance of the Ni_3_Fe LDH, NiFe E5, and NiFe ER5. (a) *iR*-corrected polarization curves at a scanning rate of 5 mV/s, (b) Tafel plots, and (c) overpotentials at a current density of 10 mA/cm^2^ and Tafel slopes; (d) chronopotentiometric curves of NiFe E5 and NiFe ER5 under a constant current density of 100 mA/cm^2^; (e) Nyquist plots at an overpotential of 270 mV of the three LDH samples, glassy carbon electrode, and RuO_2_; (f) linear plots of the average current density during 0.95–1.00 V versus scanning speed; and (g–i) cyclic voltammetry curves of the three as-synthesized LDH samples at a scanning rate of 100 mV/s during the initial 20 circles. The equivalent circuit used for the fitting of EIS data is shown on the right panel of (e).

The electrocatalytic performance of these NiFe LDH nanomaterials was evaluated by coating the samples on a glassy carbon electrode. As shown in Figure 4a–c, the *iR*-corrected polarization curves and Tafel plots demonstrate that the glassy carbon electrode has an overpotential of 426 mV and a Tafel slope of 110 mV/dec; these values are greatly reduced to 295 mV and 65 mV/dec when coated with Ni_3_Fe LDH. Etching the LDHs with nitric acid at RT can further decrease the overpotential to 262 mV and the Tafel slope to 57 mV/dec, which are similar to previously reported results.^19,20^ This performance already surpasses the commercial benchmarked ruthenium oxide (RuO_2_) electrocatalyst that exhibits an overpotential of 274 mV and a Tafel slope of 72 mV/dec. Remarkably, the OER activity of NiFe ER5, synthesized by the etching-and-recrystallization method, can reach an even lower overpotential of 241 mV and lower Tafel slope of 43 mV/dec, which is among the best NiFe and CoFe LDH electrocatalysts formed using different defect engineering strategies reported elsewhere (Table S4). Other methods report a smaller overpotential close or lower than 200 mV, yet most of them are based on doping NiFe LDHs with other elements^38^ or growing LDHs on conducting materials.^39^ The addition of 5 mL of 0.9 M nitric acid was found to be the optimal acid treatment; the use of less acid led to fewer defects and more acid resulted in the overetching of LDH structures and the formation of significant amounts of a nano-Fe_3_O_4_ impurity; both factors have a negative effect on the OER performance (Figures S18 and S19, Tables S1 and S2). We noted that Fe_3_O_4_ (NiFe ER10) is inactive for the OER, presenting a low activity with an overpotential of 399 mV and a Tafel slope of 79 mV/dec (Figure S19 and Table S2). We found that hydrothermal treatment at 100 °C was the optimal recrystallization temperature, as higher temperatures produced larger Fe_3_O_4_ nanoparticles with decreased OER activity (Figure S20 and Table S3) and lower temperatures were not sufficient to balance the etching and crystal growth of the NiFe LDHs. Apart from a low overpotential and Tafel slope, NiFe ER5 also shows excellent stability, working stably for 50 h at a current density of 100 mA/cm^2^ with an applied voltage of ca. 30 mV lower than that of NiFe E5 (Figure 4d).

We employed electrochemical impedance spectroscopy (EIS) at 1.50 V (potential vs RHE) to evaluate the OER kinetics of the various samples. The EIS curves are fitted with an equivalent circuit consisting of *R*_1_ (ascribed to electrolyte resistance), *R*_2_ (ascribed to interfacial resistance), *R*_3_ (ascribed to charge transfer resistance), and CPE_1_/CPE_2_ (constant phase elements). We selected this model because it considers the existence of two arcs in the high- and low-frequency ranges, indicating the participation of multistep processes during the OER pathways. As shown in the Nyquist plots in Figure 4e and Table S5, NiFe ER5 exhibits the smallest *R*_3_ (25.6 Ω) compared with that of Ni_3_Fe LDH (66.0 Ω), RuO_2_ (54.0 Ω), and NiFe E5 (28.9 Ω), demonstrating that NiFe ER5 has the fastest charge transfer for the water oxidation (Figure 4e). Electrochemical double-layer capacitance was applied to evaluate the effect of the electrochemically active surface area (ECSA) (Figure S21). As shown in Figure 4f, the fitted double-layer capacitance of NiFe ER5 is 651 μF/cm^2^, lower than that of Ni_3_Fe LDH (675 μF/cm^2^) and NiFe E5 (1196 μF/cm^2^), revealing that the high OER activity of NiFe ER5 is not due to an active surface area. This implies that each active site of NiFe ER5 has higher activity than those of Ni_3_Fe LDH and NiFe E5, which will be explored in the theoretical calculation section. Changes in the active sites were detected by cyclic voltammetry (CV) during the activation stage (initial 20 cycles). As shown in Figure 4g, Ni_3_Fe LDH shows a broad Ni^2+^ oxidation peak mixed with the OER region, which is ascribed to its low crystallinity. The current density at an applied voltage of 1.60 V (vs RHE) gradually increases from 25 mA/cm^2^ on the first cycle to reach a stable value of 34 mA/cm^2^ after the 10th cycle, suggesting that some additional sites are activated during the CV scanning. In the case of NiFe E5, it reaches a highest current density of 53 mA/cm^2^ at the first cycle that gradually drops to 48 mA/cm^2^, suggesting that it has more exposed sites at the beginning, and some sites were unstable and unactivated during the OER process (Figure 4h). By contrast, the Ni^2+^ oxidation peak of NiFe ER5 is more distinguishable because of its higher crystallinity. It shows a high current density of 53 mA/cm^2^ in the initial cycle, which increases to 67 mA/cm^2^ after 20 cycles, indicating that it not only has many initially available exposed sites but also has some hidden sites that can be activated during the CV scanning (Figure 4i). These findings suggest that traditional etching methods can produce LDHs with significant surface vacancies, presumable on both platelet surfaces and edges. However, this approach clauses significant damage to the layered structure; some active sites become unstable and rapidly deactivated at the beginning of the OER process and eventually cannot contribute to the OER activity. Instead, our etching-and-recrystallization approach provides NiFe LDHs with abundant surface vacancies and a more stable layered framework, offering abundant initial active sites and additional internal sites, which can be activated during the OER process, thus providing an overall superior OER electrocatalytic performance.

### DFT Simulation Analysis

**Figure 5:**
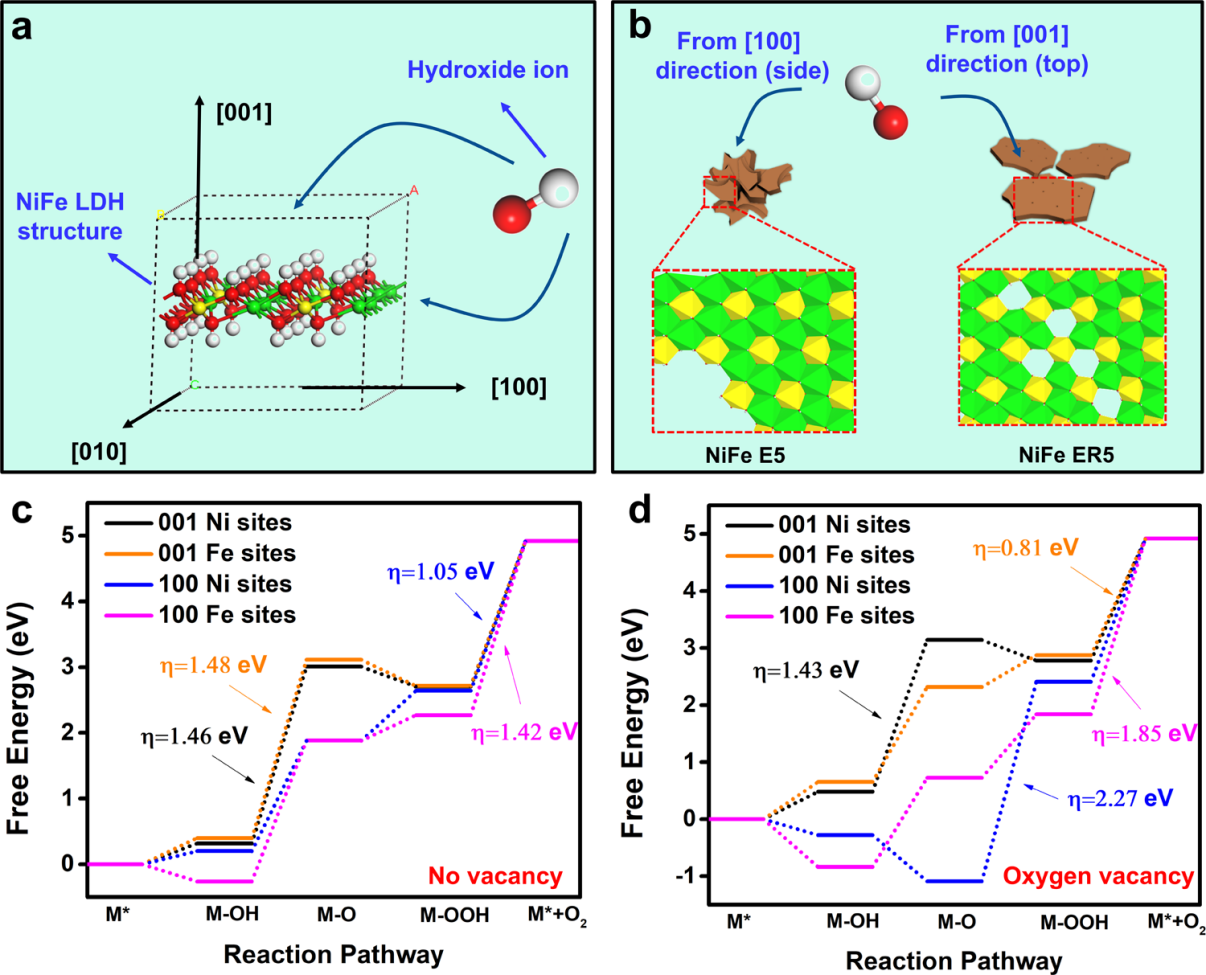
DFT simulation of the OER process in the pristine and defect-containing NiFe LDHs. (a) Schematic showing the attack of a hydroxide ion from either the top [001] direction or the lateral [100] direction of the NiFe LDH (H: white, O: red, Ni: green, and Fe: yellow); (b) schematic showing the main attack direction of a hydroxide ion in the NiFe E5 and NiFe ER5; and calculated free energy diagram for the OER process on the top [001] direction and the lateral [100] direction of (c) pristine Ni_3_Fe LDH and (d) NiFe LDHs with oxygen vacancies. The numbers indicated refer to the theoretical overpotential in each case.

To further elucidate the enhanced OER performance in the LDHs obtained by an etching-and-recrystallization method, first-principles calculations based on density functional theory (DFT) methods were performed.^40,41^ As schematically shown in Figure 5a, the OER process, starting from the attack of the hydroxide ions on the catalysts, can happen either on the top [001] direction or the lateral [100] direction of the 2D LDH structure. The LDH after etching at RT is significantly defective and fragmented, thus showing a higher proportion of lateral surface and a higher inclination for performing the OER process on the [100] direction. In contrast, the LDH after etching and recrystallization has a larger CDL due to the hydrothermal recrystallization, resulting in a higher exposure of an (001) face for the OER process (Figure 5b). We first calculated the change of reaction free energy based on the adsorbate evolution mechanism, which involves four elementary steps in alkaline conditions: M*, M–OH, M–O, and M–OOH.^10^ The theoretical overpotential is determined by the energy step showing the highest free energy increase (see the Methods section). Four reaction routes including two reaction directions ([001] and [100] directions) and two active sites (Fe and Ni sites) were considered for the calculation. For the pristine LDHs without any vacancies, the theoretical overpotentials on Ni sites are 1.46 V for the [001] direction and 1.05 V for the [100] direction, lower than those on Fe sites, demonstrating that Ni sites are more active than the Fe sites, which is consistent with previous reports (Figures 5c, S22, and S23).^20,42^ Additionally, the theoretical overpotential of the routes on the lateral [100] direction is lower than that on the [001] direction on both Ni and Fe sites, meaning that smaller and thicker LDHs with more exposure of lateral surfaces would be beneficial for the catalysis. This finding based on the LDH without any vacancies seems to conflict with what we found in the NiFe ER5 (smaller and thinner) and for ultrathin LDHs reported elsewhere.^25,43^ However, if the defect effect is considered, particularly the oxygen vacancies, it could significantly change the electronic structure and reaction kinetics of the catalysts. As shown in Figures 5d, S24, and S25, the minimum theoretical overpotential is changed from 1.05 V in the pristine LDHs to 0.81 V in the LDHs containing oxygen vacancies, consistent with the improved catalytic activity caused by defective NiFe LDHs. Most importantly, theoretical overpotentials for routes via the top [001] direction are more advantageous than those via the [100] direction. This result is consistent with the enhanced OER activity of ultrathin structures and helps to explain the superior activity of NiFe ER5 relative to NiFe E5 since NiFe ER5 has a larger platelet diameter and thus more exposure of the top (001) surface. The change in favor of the OER along [100] to along the [001] direction is mainly due to the oxygen vacancies modifying the electronic structure of the catalysts. This results in a strong adsorption of hydroxide ions on the [100] direction, making the remaining elementary steps more energetic to overcome the OER barrier. These DFT calculations suggest that the superior OER activity of NiFe ER5 is mainly due to its defect-abundant structure along the basal (001) surface.

## Conclusions

We have developed an etching-and-recrystallization method to prepare defect-rich NiFe LDH nanoplatelets with superior OER activity. Compared to existing LDHs obtained by traditional etching methods, our nanoplatelets are free-standing rather than being agglomerated, having a larger platelet diameter and a relatively high degree of crystallinity, with more abundant surface oxygen and metal vacancies along the basal (001) plane. The etching process allows the introduction of abundant surface vacancies in the material, leading to high initial catalytic activity, while the hydrothermal recrystallization process ensures a relatively crystalline 2D structure, which makes the initial active sites more stable and other internal sites can be activated during the OER process; both processes contribute to the enhancement in OER performance. To date, these NiFe LDH nanoplatelets exhibit an overpotential of 241 mV and lowest Tafel slope of 43 mV/dec for the OER process; this currently places them as the best transition metal-based LDH electrocatalysts prepared using defect engineering methods. Physical characterization and DFT simulation suggest that the enhanced electrocatalytic activity of the NiFe LDH nanoplatelets results mainly from their abundant defects along the basal (001) surface. This strategy could be a promising method to design other defect-abundant catalysts (e.g., photocatalysts), thus paving the way for the synthesis of other high-performance catalysts.

## Supporting Information Available

SEM images, XRD patterns, Raman spectra, BET analyses, XPS spectra, and XAS analyses of various LDH samples and optimized structures of pristine and defective LDH models (PDF)
