# Supplementary material for: Intrabasal Plane Defect Formation in NiFe Layered Double Hydroxides Enabling Efficient Electrochemical Water Oxidation
Source: ACS Appl Mater Interfaces. 2023 Nov 10;15(46):53815–26. doi: 10.1021/acsami.3c11651 (PMC10685352; doi:10.1021/acsami.3c11651)
Supplement: Supplementary file 1 — am3c11651_si_001.pdf [file am3c11651_si_001.pdf]

Supporting Information

Intrabasal Plane Defect Formation in NiFe Layered Double Hydroxides Enabling Efficient Electrochemical Water Oxidation

Xiaopeng Huang[a,b], Keon-Han Kim[b], Haeseong Jang[c], Xiaonan Luo[d], Jingfang Yu[e,f], Zhaoqiang Li[g], Zhimin Ao[h,i], Junxin Wang[b,j], Hao Zhang[b], Chunping Chen[b]* and Dermot O'Hare[b]*

[a] *Department of Chemistry, Faculty of Arts and Sciences, Beijing Normal University, Zhuhai, 519087, China*

[b] *Chemistry Research Laboratory, Department of Chemistry, University of Oxford, 12 Mansfield Road, Oxford, OX1 3TA UK*

[c] *Beamline Research Division, Pohang Accelerator Laboratory (PAL), Pohang, 37673, Republic of Korea*

[d] *Department of Materials, University of Oxford, 16 Parks Road, OX1 3PH, Oxford, UK*

[e] *Engineering Research Center of NanoGeomaterials of Ministry of Education, China University of Geosciences, Wuhan 430074, China*

[f] *Faculty of Materials Science and Chemistry, China University of Geosciences, Wuhan 430074, China*

[g] *Laboratory of Beam Technology and Energy Materials, Advanced Institute of Natural Sciences, Beijing Normal University, Zhuhai, 519087, China*

[h] *Institute of Environmental Health and Pollution Control, School of Environmental Science and Engineering, Guangdong University of Technology, Guangzhou 510006, China*

[i] *Advanced Interdisciplinary Institute of Environment and Ecology, Beijing Normal University, Zhuhai 519087, PR China*

[j] *Department of Materials Science and Metallurgy, University of Cambridge, 27 Charles Babbage Road, Cambridge CB3 0FS, United Kingdom*

*Corresponding Authors: chunping.chen@chem.oc.ac.uk; dermot.ohare@chem.ox.ac.uk

Table of Contents

1. Figure S1 SEM and optical images of NiFe Control, NiFe E1, NiFe E2, NiFe E5 and NiFe E7.5

2. Figure S2 SEM and optical images of NiFe ER0, NiFe ER1, NiFe ER2, NiFe ER5, NiFe ER7.5 and NiFe ER10

3. Figure S3 SEM and optical images of NiFe Control, NiFe E5 (25 ℃), NiFe E5 (50 ℃), NiFe ER5 (100 ℃), NiFe ER5 (150 ℃) and NiFe ER5 (200 ℃)

4. Figure S4 High-resolution scanning transmission electron microscope (STEM) images of NiFe ER5 (100 ℃)

5. Figure S5 XRD patterns of NiFe Control, NiFe E1, NiFe E2, NiFe E5 and NiFe E7.5

6. Figure S6 XRD patterns of NiFe Control, NiFe ER0, NiFe ER1, NiFe ER2, NiFe ER5, NiFe ER7.5 and NiFe ER10

7. Figure S7 XRD patterns of NiFe Control, NiFe E5 (25 ℃), NiFe E5 (50 ℃), NiFe ER5 (100 ℃), NiFe ER5 (150 ℃) and NiFe ER5 (200 ℃)

8. Figure S8 Raman spectra of NiFe Control, NiFe E1, NiFe E2, NiFe E5, NiFe E7.5 and NiFe E10

9. Figure S9 Raman spectra of sodium carbonate, nickel nitrate , NiFe Control, NiFe ER0, NiFe ER1, NiFe ER2, NiFe ER5, NiFe ER7.5 and NiFe ER10

10. Figure S10 Raman spectra of NiFe Control, NiFe E5 (25 ℃), NiFe E5 (50 ℃), NiFe ER5 (100 ℃), NiFe ER5 (150 ℃) and NiFe ER5 (200 ℃)

11. Figure S11 Thermogravimetric analysis (TGA) of NiFe Control, NiFe ER0, NiFe ER1, NiFe ER2, NiFe ER5, NiFe ER7.5 and NiFe ER10

12. Figure S12 The BET surface area, average pore diameter and cumulative pore volume of NiFe Control, NiFe E5 and NiFe ER5

13. Figure S13 The pH change of various NiFe LDHs before and after post-synthesis treatment

14. Figure S14 Deconvolution of the Ni 2p XPS spectra of NiFe Control, NiFe E5 and NiFe ER5

15. Figure S15 Deconvolution of the Fe 2p XPS spectra of NiFe Control, NiFe E5 and NiFe ER5

16. Figure S16 Deconvolution of the O 1s XPS spectra of NiFe Control, NiFe E5 and NiFe ER5

17. Figure S17 Fe K-edge XANES and EXAFS analysis of NiFe Control, NiFe E5 and NiFe ER5

18. Figure S18 Polarization curves and Tafel plots of NiFe Control, NiFe E1, NiFe E2, NiFe E5 and NiFe E7.5

19. Figure S19 Polarization curves and Tafel plots of NiFe Control, NiFe ER0, NiFe ER1, NiFe ER2, NiFe ER5, NiFe ER7.5 and NiFe ER10

20. Figure S20 Polarization curves and Tafel plots of NiFe Control, NiFe E5 (25 °C) and NiFe E5 (50 °C), NiFe ER5 (100 °C), NiFe ER5 (150 °C) and NiFe ER5 (200 °C)

21. Figure S21 Cyclic voltammetry curves of NiFe Control, NiFe E5 and NiFe ER5

22. Figure S22 Optimised structures of the (001) slab of pristine NiFe LDHs

23. Figure S23 Optimised structures of the (100) slab of pristine NiFe LDHs

24. Figure S24 Optimised structures of the (001) slab of NiFe LDHs containing oxygen vacancies

25. Figure S25 Optimised structures of the (100) slab of NiFe LDHs containing oxygen vacancies

26. Table S1 The lattice parameters, crystal domain sizes, OER overpotentials at a current density of 10 mA/cm^2, and Tafel slopes of Ni_3Fe LDH and NiFe Ey samples

27. Table S2 The lattice parameters, crystal domain sizes, OER overpotentials at a current density of 10 mA/cm^2, and Tafel slopes of Ni_3Fe LDH and NiFe ERy samples

28. Table S3 The lattice parameters, crystal domain sizes, OER overpotentials at a current density of 10 mA/cm^2, and Tafel slopes of Ni_3Fe LDH, NiFe E5 at 25 °C

and 50 ℃, NiFe ER5 samples at 100 ℃, 150 ℃ and 200 ℃

29. Table S4 Summary of different strategies to synthesise transition metals-based LDH electrocatalysts with vacancies and their OER catalytic activity

30. Table S5. The fitted resistance results from Nyquist plots in Fig. 4e

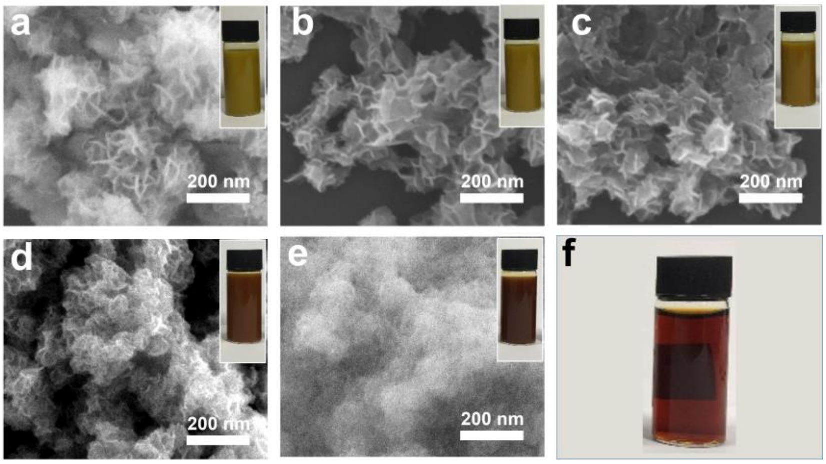

Figure S1. (a-e) SEM and optical images of (a) Ni_3Fe LDH, (b) NiFe E1, (c) NiFe E2, (d) NiFe E5, (e) NiFe E7.5, respectively. The insets are the optical images of the corresponding LDHs in water. (f) Optical image of NiFe E10, obtained after etching with 10 mL nitric acid, showing clear salt solution without precipitates.

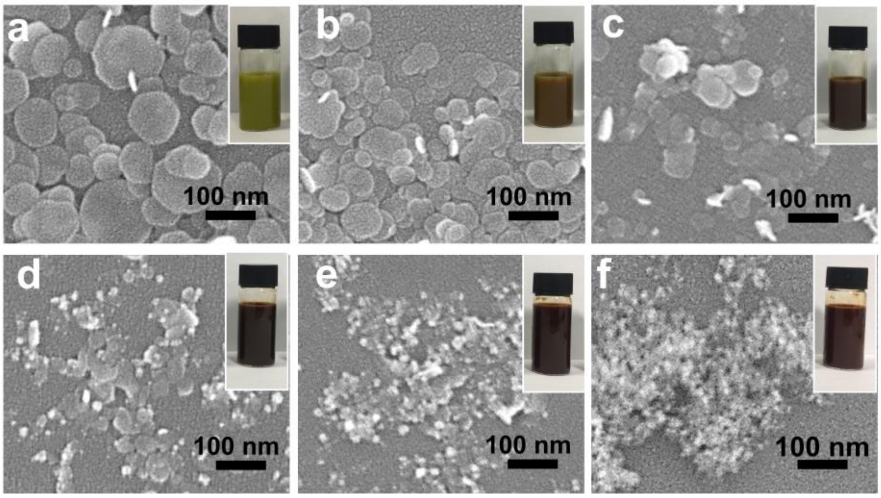

Figure S2. (a-f) SEM and optical images of (a) NiFe ER0, (b) NiFe ER1, (c) NiFe ER2, (d) NiFe ER5, (e) NiFe ER7.5, and (f) NiFe ER10, respectively. The insets are the optical images of the corresponding LDHs in water.

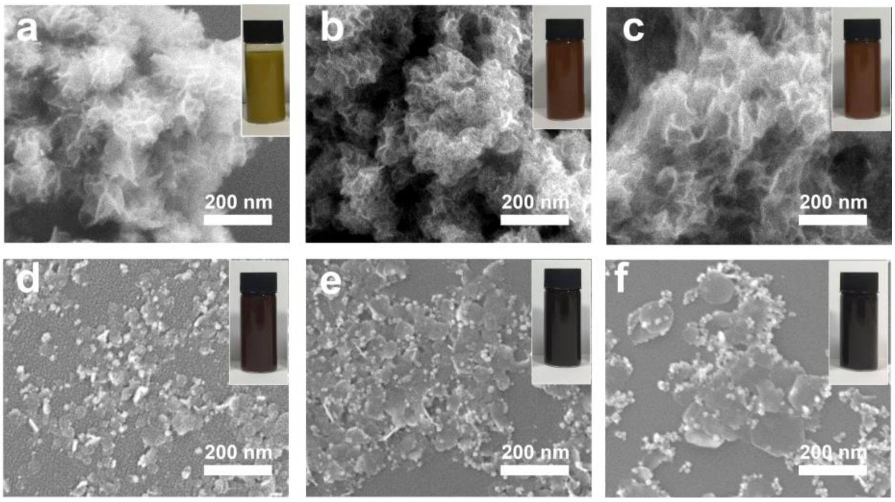

Figure S3. (a-f) SEM and optical images of (a) Ni_3Fe LDH, (b) NiFe E5 (25 ℃), (c) NiFe E5 (50 ℃), (d) NiFe ER5 (100 ℃), (e) NiFe ER5 (150 ℃), (f) and NiFe ER5 (200 ℃). The insets are the optical images of the corresponding LDHs in water.

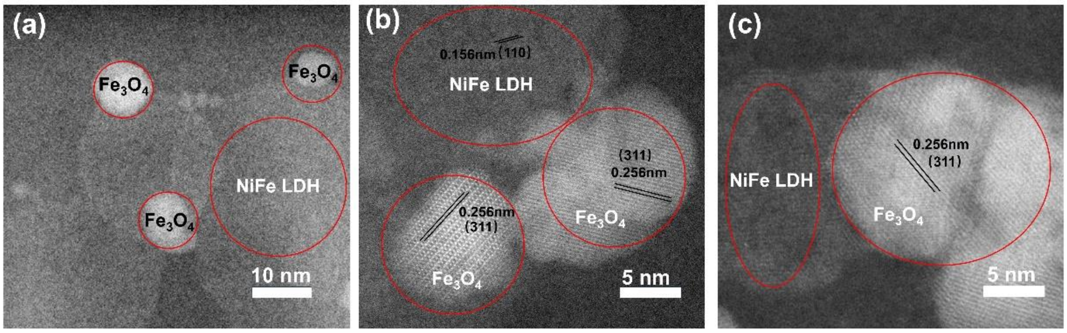

Figure S4. (a-c) High-resolution scanning transmission electron microscope (STEM) images of NiFe ER5 (100 ℃).

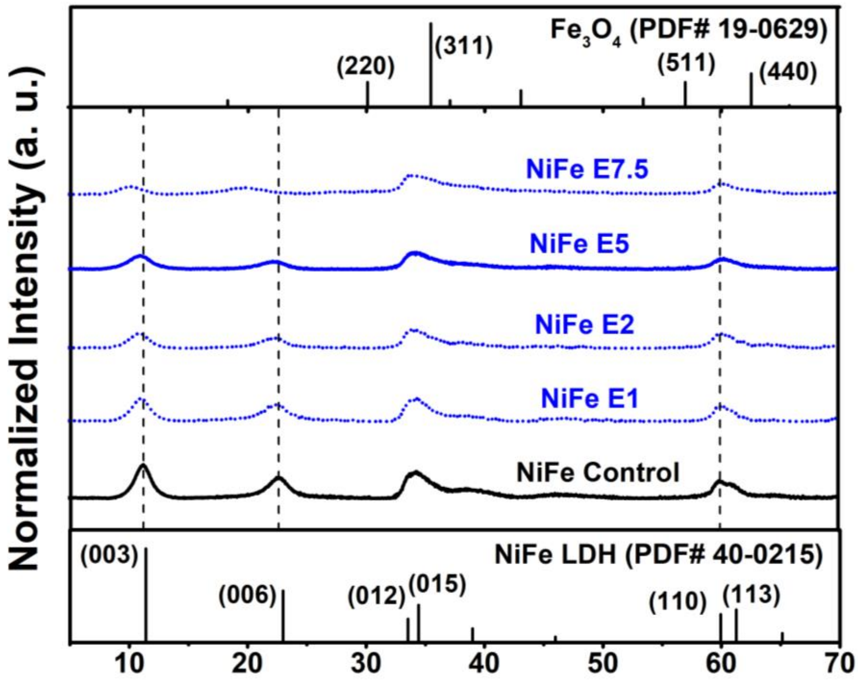

Figure S5. XRD patterns of Ni_3Fe LDH, NiFe E1, NiFe E2, NiFe E5 and NiFe E7.5, respectively.

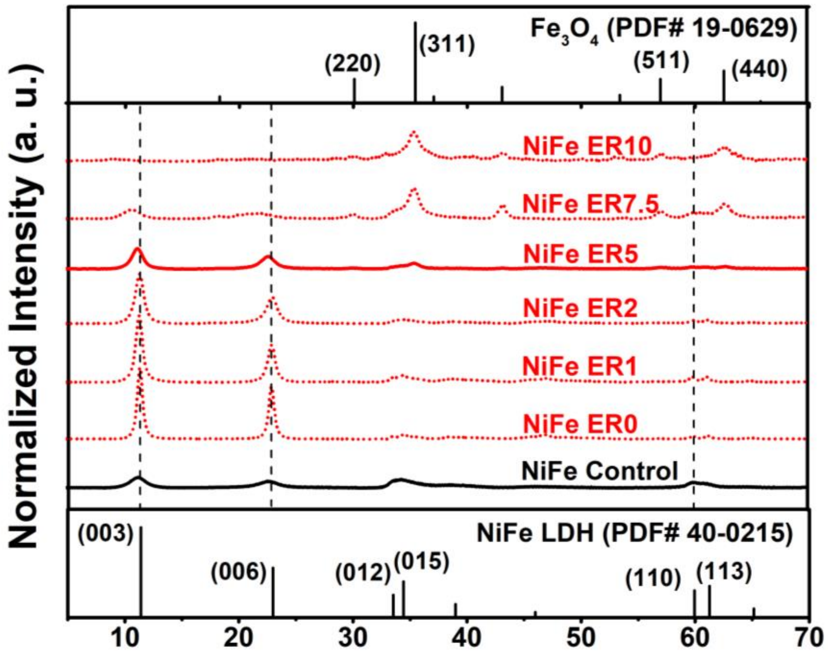

Figure S6. XRD patterns of Ni_3Fe LDH, NiFe ER0, NiFe ER1, NiFe ER2, NiFe ER5, NiFe ER7.5 and NiFe ER10, respectively.

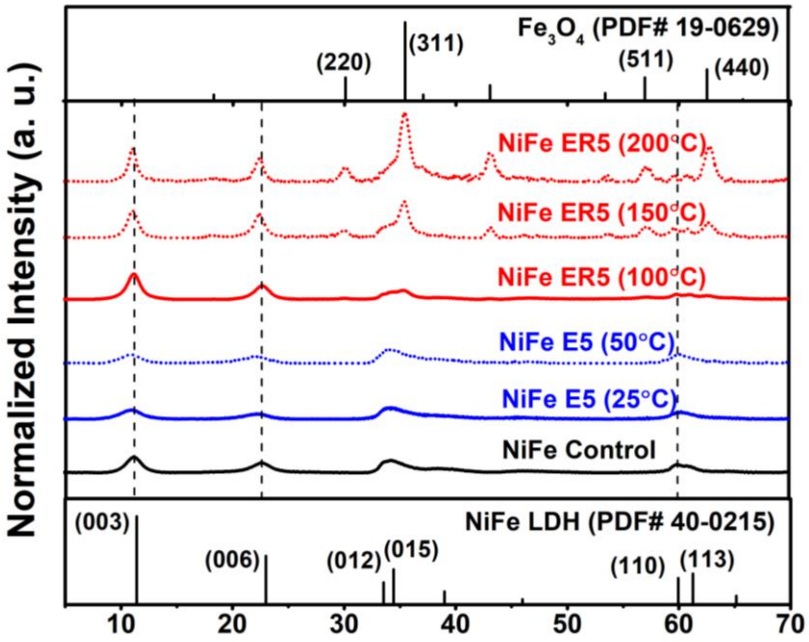

Figure S7. XRD patterns of Ni_3Fe LDH, NiFe E5 (25 °C), NiFe E5 (50 °C), NiFe ER5 (100 °C), NiFe ER5 (150 °C) and NiFe ER5 (200 °C), respectively.

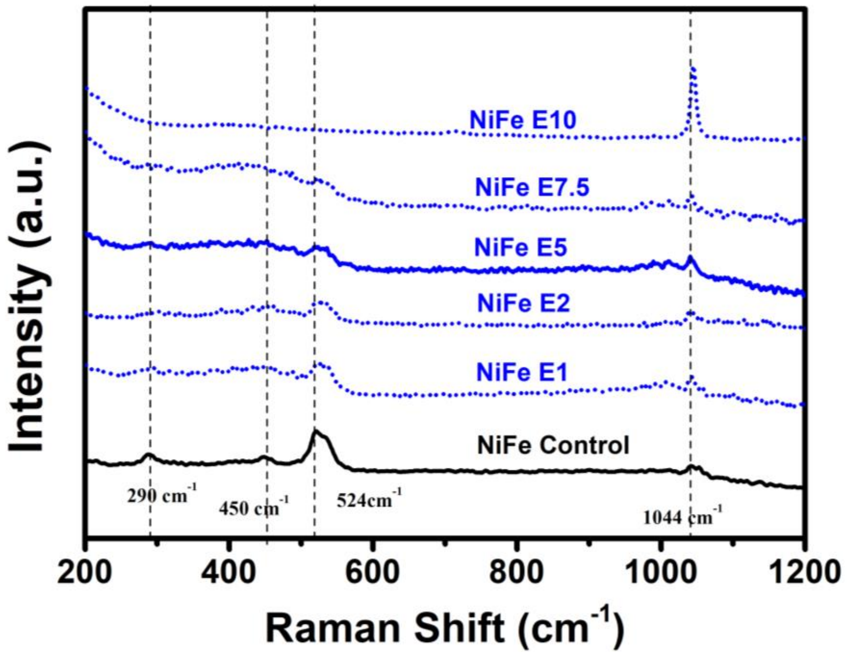

Figure S8. Raman spectra of Ni_3Fe LDH, NiFe E1, NiFe E2, NiFe E5, NiFe E7.5 and NiFe E10, respectively.

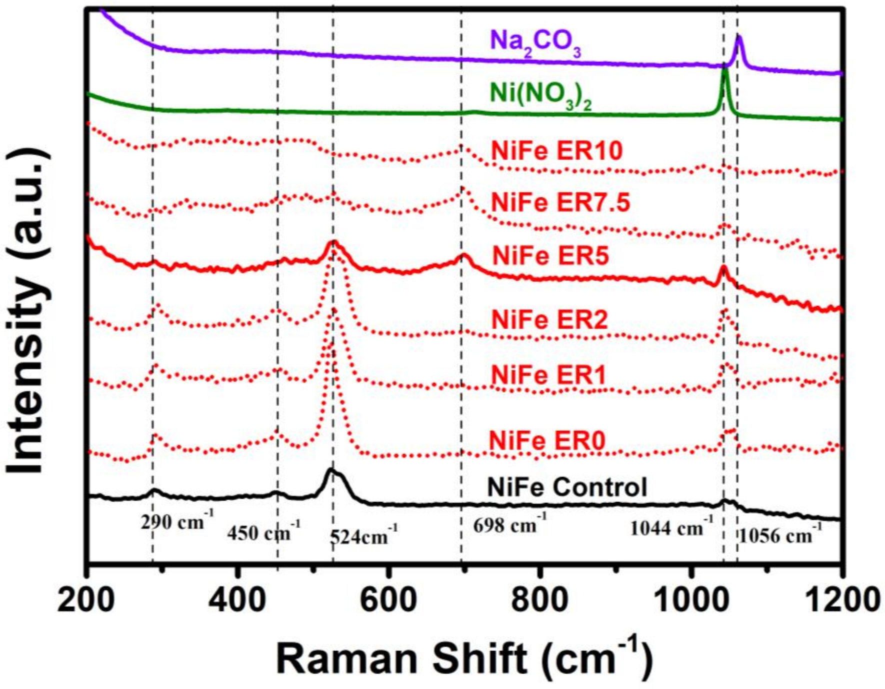

Figure S9. Raman spectra of sodium carbonate (Na_2CO_3), nickel nitrate {$Ni(NO_3)_2$}, Ni_3Fe LDH, NiFe ER0, NiFe ER1, NiFe ER2, NiFe ER5, NiFe ER7.5 and NiFe ER10, respectively.

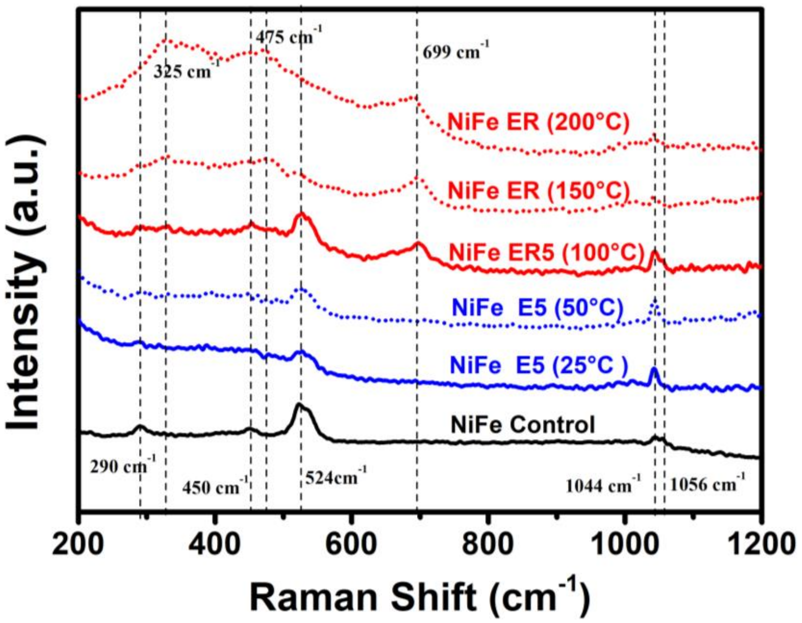

Figure S10. Raman spectra of Ni_3Fe LDH, NiFe E5 (25 °C) and NiFe E5 (50 °C), NiFe ER5 (100 °C), NiFe ER5 (150 °C) and NiFe ER5 (200 °C), respectively.

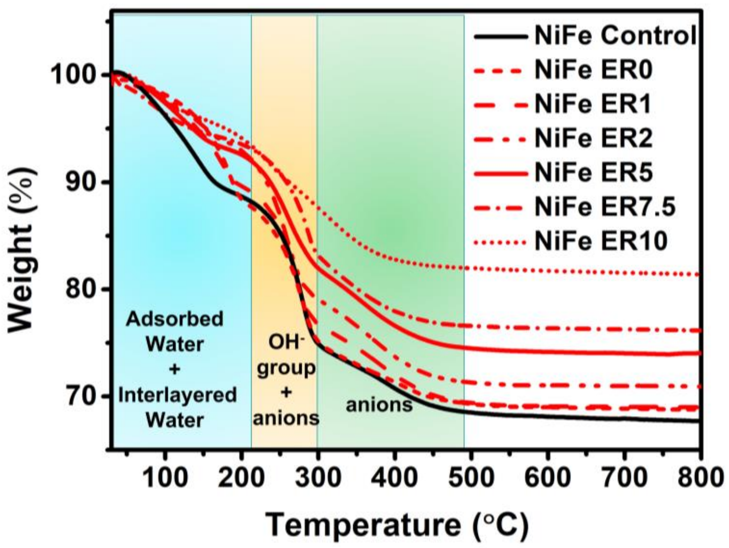

Figure S11. Thermogravimetric analysis data (TGA) for Ni_3Fe LDH, NiFe ER0, NiFe ER1, NiFe ER2, NiFe ER5, NiFe ER7.5 and NiFe ER10.

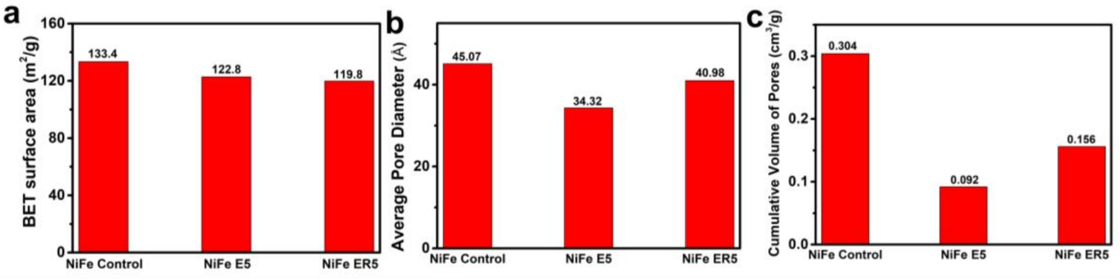

Figure S12. (a) The N_2 surface area using BET model, (b) average pore diameter and (c) cumulative pore volume for Ni_3Fe LDH, NiFe E5 and NiFe ER5.

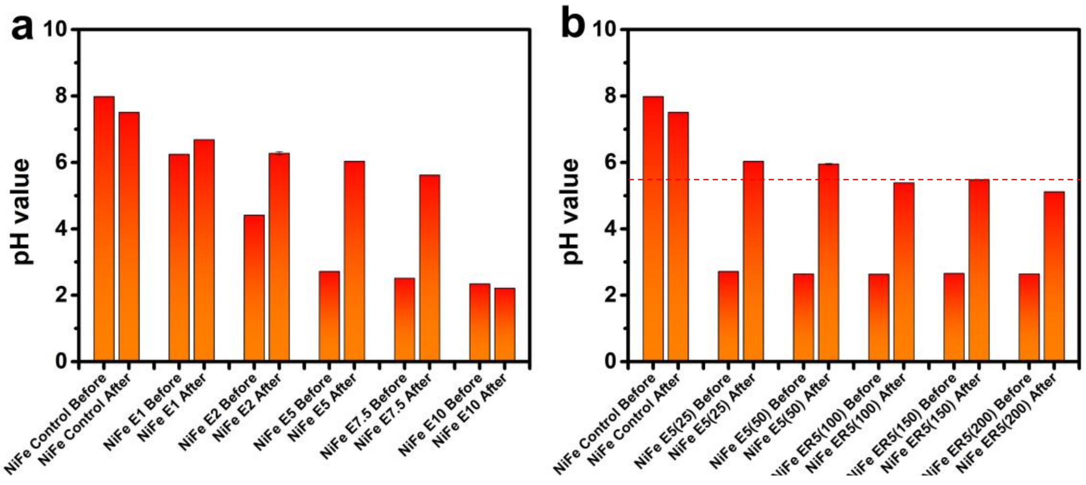

Figure S13. The pH change of various Ni_3Fe LDHs before and after post-synthesis treatment. (a) The pH change before and after aging for 20h at RT for Ni_3Fe LDH, NiFe E1, NiFe E2, NiFe E5, NiFe E7.5

and NiFe E10 respectively. (b) The pH change before and after aging for 20h at different temperatures for Ni_3Fe LDH, NiFe E5 (25) and NiFe E5 (50), NiFe ER5 (100), NiFe ER5 (150), NiFe ER5 (200) respectively. The pH change reflects the concentration of [H+] or [OH-] in the solution. An increase in pH signifies the etching of LDH, while a decrease indicates the growth of LDH.

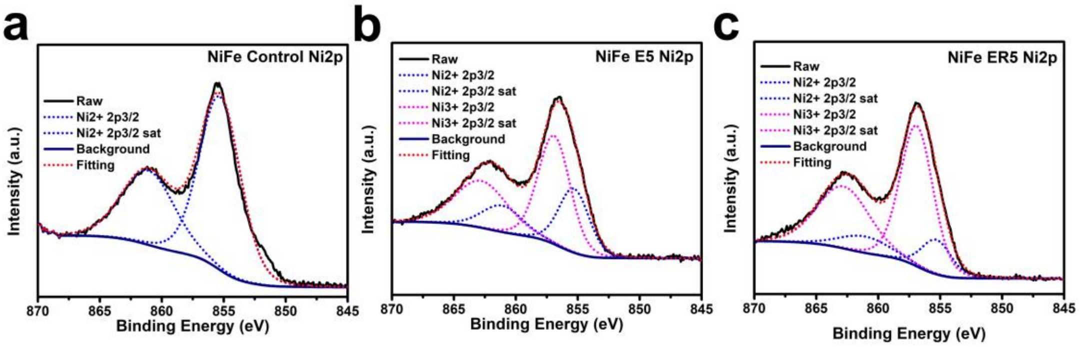

Name	Position (eV)	%At Conc
Ni2+ 2p3/2	855.3	64.43
Ni2+ 2p3/2 sat	861.1	35.53
Ni3+ 2p3/2	856.9	0.02
Ni3+ 2p3/2 sat	862.8	0.02

Name	Position (eV)	%At Conc
Ni2+ 2p3/2	855.3	21.62
Ni2+ 2p3/2 sat	861.1	11.92
Ni3+ 2p3/2	856.9	38.9
Ni3+ 2p3/2 sat	862.8	27.56

Name	Position (eV)	%At Conc
Ni2+ 2p3/2	855.3	10.53
Ni2+ 2p3/2 sat	861.1	8.49
Ni3+ 2p3/2	856.9	47.4
Ni3+ 2p3/2 sat	862.8	33.58

Figure S14. Deconvolution of the Ni 2p XPS spectra of (a) Ni_3Fe Control, (b) NiFe E5 and (c) NiFe ER5.

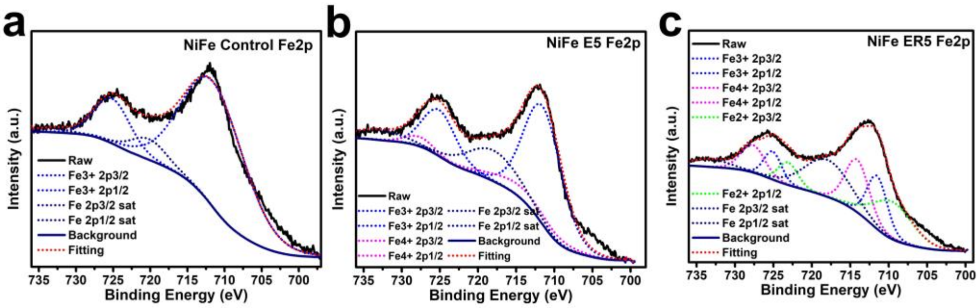

Name	Position (eV)	%At Conc
Fe3+ 2p3/2	711.5	75.66
Fe3+ 2p1/2	725.1	15.69
Fe 2p3/2 sat	720.2	7.27
Fe 2p1/2 sat	733.2	1.38

Name	Position (eV)	%At Conc
Fe3+ 2p3/2	711.5	48.99
Fe3+ 2p1/2	725.1	16.85
Fe4+ 2p3/2	714.1	8.15
Fe4+ 2p1/2	727.7	2.91
Fe 2p3/2 sat	718	17.44
Fe 2p1/2 sat	733.2	5.65

Name	Position (eV)	%At Conc
Fe3+ 2p3/2	711.5	14.52
Fe3+ 2p1/2	725.1	6.72
Fe4+ 2p3/2	714.1	16.94
Fe4+ 2p1/2	727.7	8.93
Fe2+ 2p3/2	709.5	19.89
Fe2+ 2p1/2	723.1	5.99
Fe 2p3/2 sat	718	23
Fe 2p1/2 sat	733.2	4.01

Figure S15. Deconvolution of the Fe 2p XPS spectra of (a) Ni_3Fe LDH, (b) NiFe E5 and (c) NiFe ER5.

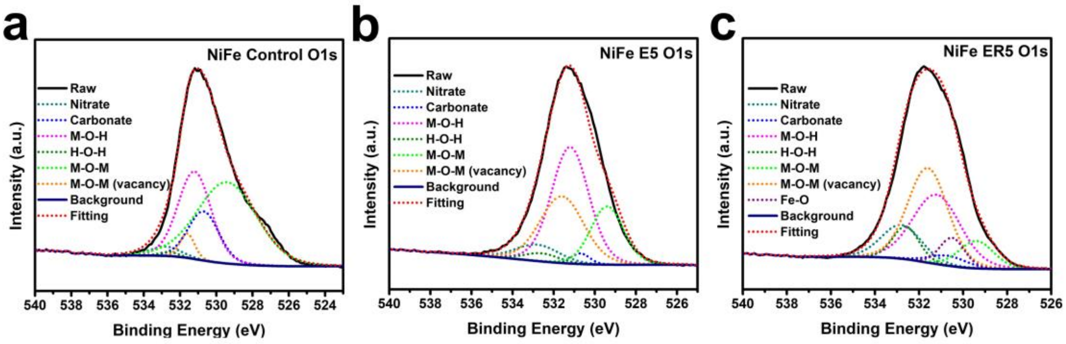

Name	Position (eV)	%At Conc
Nitrate	532.8	2.9
Carbonate	530.7	15.27
M-O-H	531.2	29.04
H-O-H	532.6	0.78
M-O-M	529.4	48.38
M-O-M (vacancy)	531.6	3.63

Name	Position (eV)	%At Conc
Nitrate	532.8	6.88
Carbonate	530.7	1.93
M-O-H	531.2	41.91
H-O-H	532.6	2.72
M-O-M	529.4	18.52
M-O-M (vacancy)	531.6	28.05

Name	Position (eV)	%At Conc
Nitrate	532.8	11.93
Carbonate	530.7	3.3
M-O-H	531.2	29.19
H-O-H	532.6	7.62
M-O-M	529.4	8.38
M-O-M (vacancy)	531.6	33.32
Fe-O	530.5	6.27

Figure S16. Deconvolution of the O 1s XPS spectra of (a) Ni_3Fe LDH, (b) NiFe E5 and (c) NiFe ER5.

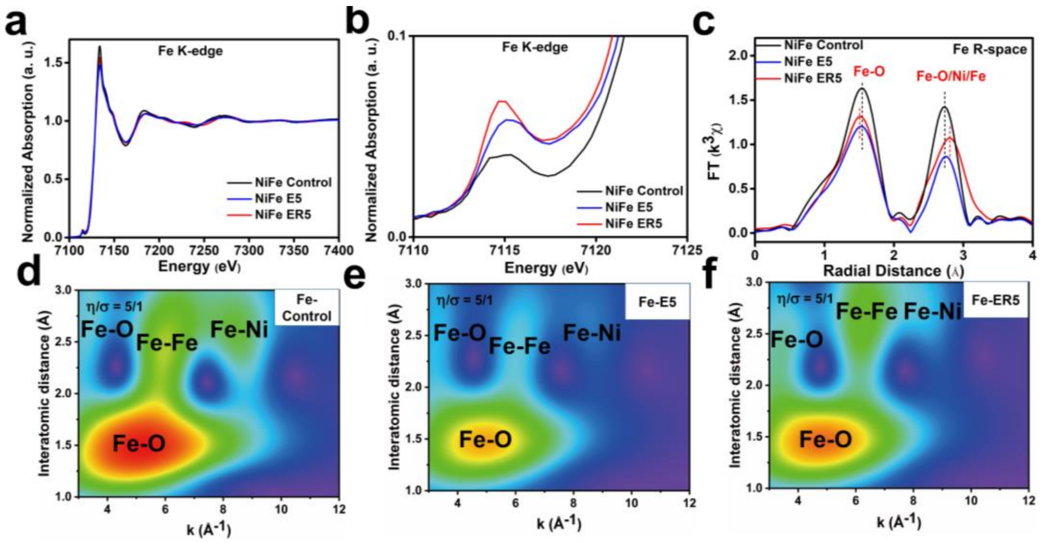

Figure S17. Fe K-edge XANES and EXAFS analysis of Ni_3Fe LDH, NiFe E5 and NiFe ER5. (a, b) XANES spectra of Fe K-edge in a full (a) and an enlarged view (b), (c) k^3-weighted Fourier-transformed Fe K-edge EXAFS data of the three samples, and (d-f) wavelet-transformed Fe K-edge EXAFS analysis of NiFe Control (d), NiFe E5 (e) and NiFe ER5 (f).

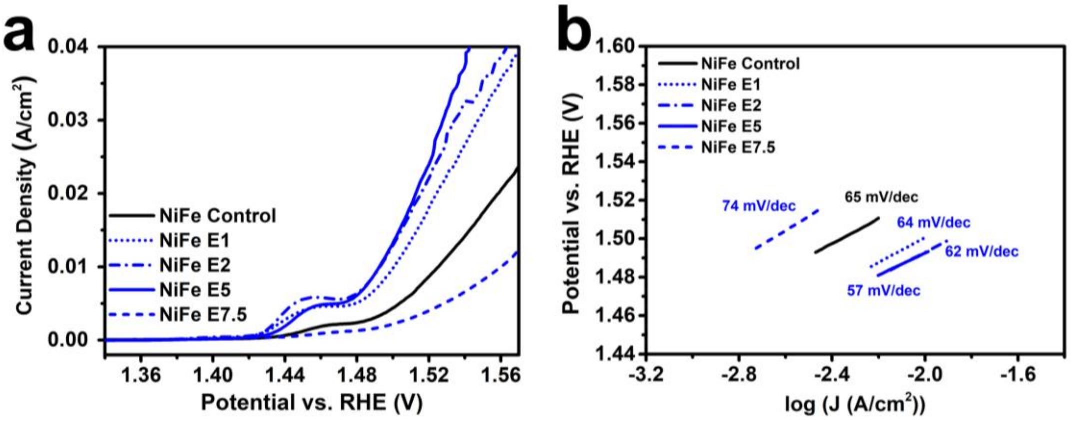

Figure S18 (a) iR-corrected polarization curves and (b) Tafel plots of Ni_3Fe LDH, NiFe E1, NiFe E2, NiFe E5 and NiFe E7.5.

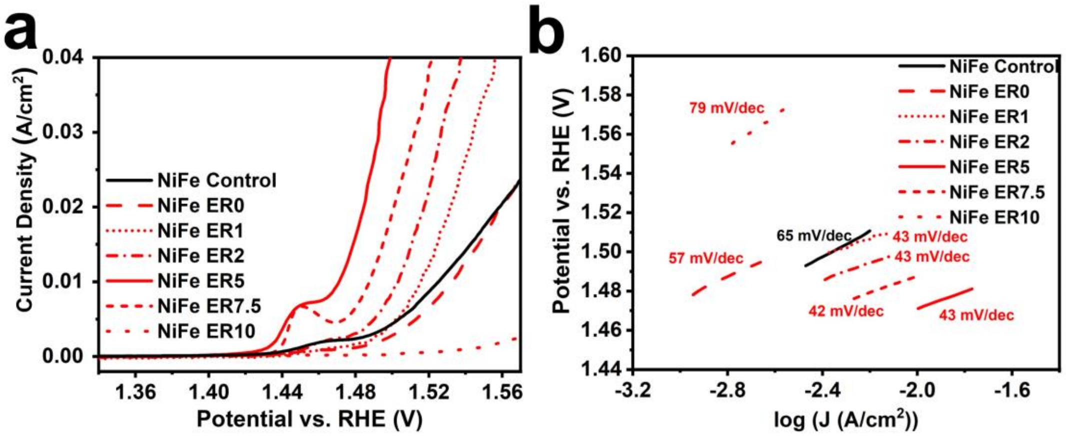

Figure S19. (a) iR-corrected polarization curves and (b) Tafel plots of Ni_3Fe LDH, NiFe ER0, NiFe ER1, NiFe ER2, NiFe ER5, NiFe ER7.5 and NiFe ER10.

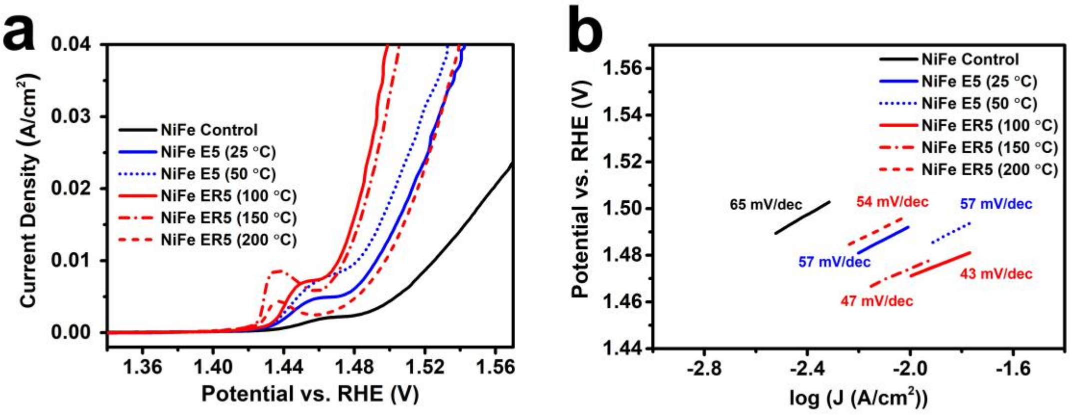

Figure S20. (a) iR-corrected polarization curves and (b) Tafel plots of Ni_3Fe LDH, NiFe E5 (25 ℃) and NiFe E5 (50 ℃), NiFe ER5 (100 ℃), NiFe ER5 (150 ℃) and NiFe ER5 (200 ℃).

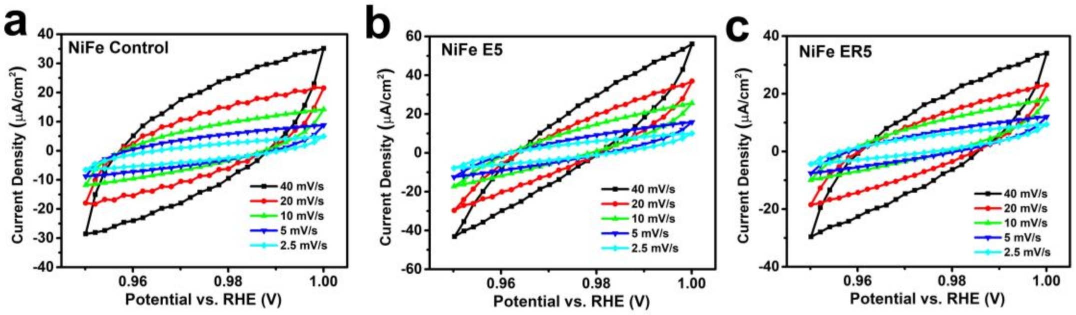

Figure S21. (a-c) Cyclic voltammetry curves of Ni_3Fe LDH (a), NiFe E5 (b) and NiFe ER5 (c) at different scanning rates during the applied voltage of 0.95 V (vs. RHE) to 1.00 V (vs. RHE), used for the calculation of double-layer electrochemical double layer capacitance.

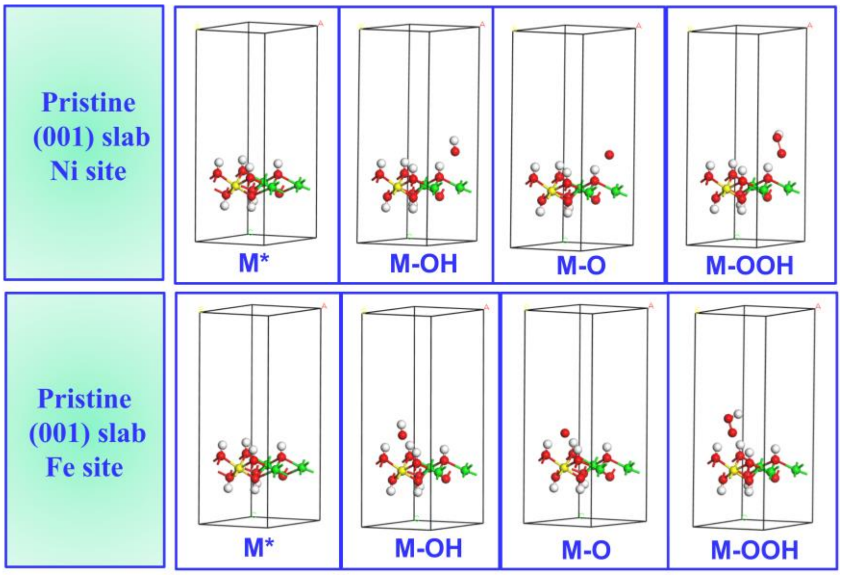

Figure S22. Optimised structures of the (001) slab of pristine Ni_3Fe LDHs before and after the adsorption of OH*, O* and OOH* intermediates on the Ni site (top) and Fe site (bottom).

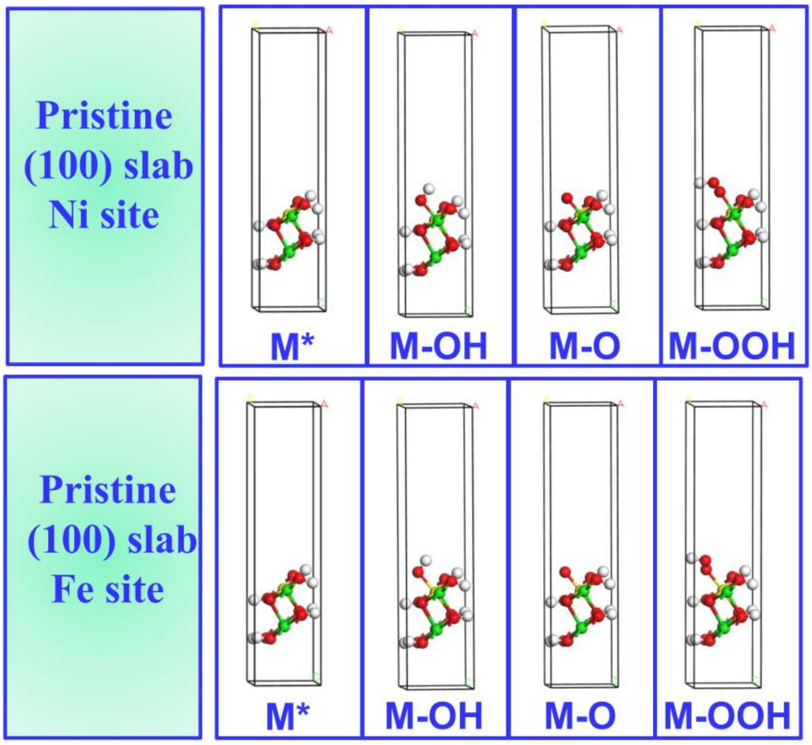

Figure S23. Optimised structures of the (100) slab of pristine Ni_3Fe LDHs before and after the adsorption of OH*, O* and OOH* intermediates on the Ni site (top) and Fe site (bottom).

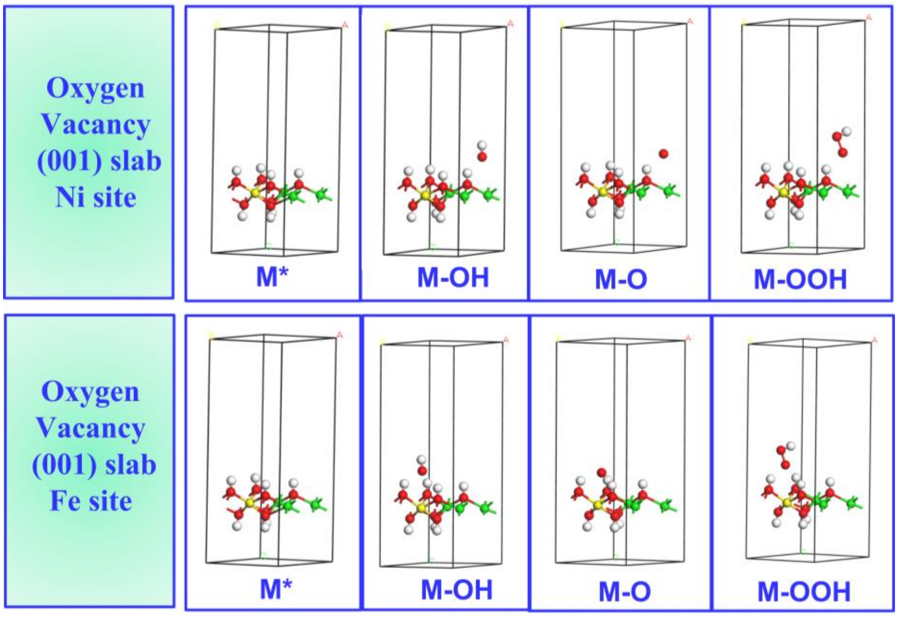

Figure S24. Optimised structures of the (001) slab of Ni_3Fe LDHs containing oxygen vacancies before and after the adsorption of OH*, O* and OOH* intermediates on the Ni site (top) and Fe site (bottom).

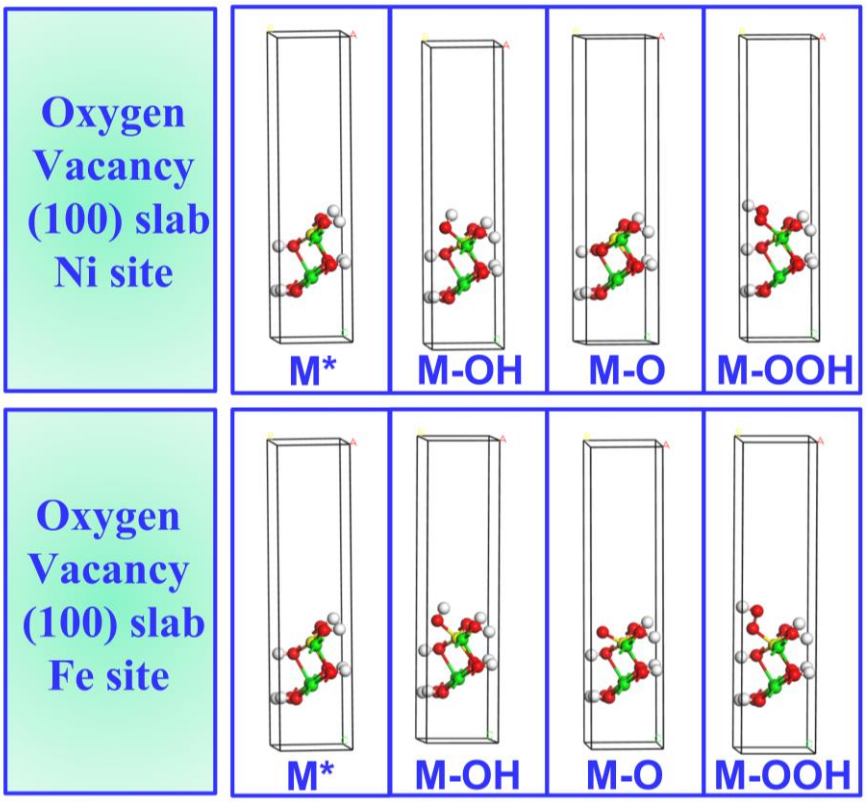

Figure S25. Optimised structures of the (100) slab of Ni_3Fe LDHs containing oxygen vacancies before and after the adsorption of OH*, O* and OOH* intermediates on the Ni site (top) and Fe site (bottom).

Table S1. The lattice parameters, crystal domain lengths, OER overpotentials at a current density of 10 mA/cm^2, and Tafel slopes of Ni_3Fe LDH and NiFe E*y* samples.

	**Lattice parameter (nm)**		**Crystal domain length (nm)**		**Overpotential at 10 mA/cm^2 (mV)**	**Tafel slope (mV/dec)**
	a (nm)	*c* (nm)	*ab* plane	*c*-axis		
Ni_3Fe LDH	0.308	2.334	12.40	8.79	295	65
NiFe E1	0.308	2.346	11.17	8.99	271	64
NiFe E2	0.308	2.370	11.56	8.29	263	62
NiFe E5	0.306	2.379	10.82	8.34	262	57
NiFe E7.5	0.306	2.574	9.36	7.18	330	74

Table S2. The lattice parameters, crystal domain sizes, OER overpotentials at a current density of 10 mA/cm^2, and Tafel slopes of Ni_3Fe LDH and NiFe ER*y* samples.

	**Lattice parameter (nm)**		**Crystal domain length (nm)**		**Overpotential at 10 mA/cm^2 (mV)**	**Tafel slope (mV/dec)**
	a (nm)	*c* (nm)	*ab*-plane	*c*-axis		
Ni_3Fe LDH	0.308	2.334	12.40	8.79	295	65
NiFe ER0	0.308	2.316	24.73	13.15	300	57
NiFe ER1	0.308	2.319	21.55	12.74	285	43
NiFe ER2	0.308	2.316	22.86	10.79	273	43
NiFe ER5	0.308	2.346	22.69	9.62	241	43
NiFe ER7.5	0.308	2.451	18.36	8.38	258	42
NiFe ER10	-	-	-	-	399	79

Table S3. The lattice parameters, crystal domain sizes, OER overpotentials at a current density of 10 mA/cm^2, and Tafel slopes of Ni_3Fe LDH, NiFe E5 at 25 ℃ and 50 ℃, NiFe ER5 samples at 100 ℃, 150 ℃ and 200 ℃.

	**Lattice parameter (nm)**		**Crystal domain length (nm)**		**Overpotential at 10 mA/cm^2 (mV)**	**Tafel slope (mV/dec)**
	a (nm)	*c* (nm)	*ab* plane	*c*-axis		
Ni_3Fe LDH	0.308	2.334	12.40	8.79	295	65
NiFe E5 (25 ℃)	0.306	2.379	10.82	8.34	251	57
NiFe E5 (50 ℃)	0.308	2.394	10.08	8.99	251	57
NiFe ER5 (100 ℃)	0.308	2.346	22.70	9.62	241	43
NiFe ER5 (150 ℃)	0.31	2.367	41.41	9.86	244	47
NiFe ER5 (200 ℃)	0.31	2.367	-	11.42	268	54

Table S4. Summary of different strategies to synthesise transition metals-based LDH electrocatalysts with vacancies and their OER catalytic activity in a 1.0 M KOH solution.

**Catalysts**	**Vacancy type**	**Synthesis Method**	**Overpotential (mV at 10 mA/cm^2 (control)**	**Tafel Slope, mV/dec (control)**	**Reference**
NiFe LDH nanoplates	O, Ni, Fe	etching-and-recrystallization (acid)	242 (296)	43 (65)	This work
Flower-like NiFe LDHs	O, Ni, Fe	acid etching	263 (296)	57 (65)	This work
NiFe LDH nanosheets	Ni	alkaline etching	229 (266)	62.9 (76.5)	Small, 2018, 14, 1800136
NiFe LDH nanosheets	Fe	alkaline etching	245 (266)	70.0 (76.5)	Small, 2018, 14, 1800136
NiFe LDH arrays	O	flame etching	250 (330)	69 (127)	Small Methods 2018, 2, 1800083
CoFe LDH nanosheets	O, Co, Fe	Ar plasma etching	266 (321)	36 (57)	Angew. Chem. Int. Ed. 2017, 56, 5867
CoFe LDH nanosheets	O, Co, Fe	water plasma etching	290 (332)	36 (52)	Adv. Mater, 2017, 29, 1701546
CoFe LDH nanosheets	O, Co, Fe	acid etching	300 (346)	41 (78)	Chem. Commun., 2017, 53, 11778
NiFe LDH nanosheets	Ni, Fe	acid etching	308 (342)	50 (80)	ChemSusChem 2020, 13, 811

Table S5. The fitted resistance results from Nyquist plots in Fig. 4e.

	**R_1 (Ω)**	**R_2 (Ω)**	**R_3 (Ω)**
RuO_2	23.1	27.2	54.0
NiFe Control	18.6	52.7	66.0
NiFe E5	17.7	19.2	28.9
NiFe ER5	18.7	15.8	25.6
